# Supplementary material for: Immuno-protective vesicle-crosslinked hydrogel for allogenic transplantation
Source: Nat Commun. 2024 Jun 18;15:5176. doi: 10.1038/s41467-024-49135-x (PMC11189436; doi:10.1038/s41467-024-49135-x)
Supplement: Supplementary file 1 — Supplementary Information [file 41467_2024_49135_MOESM1_ESM.pdf]

Supplementary Information

Immuno-protective vesicle-crosslinked hydrogel for allogenic transplantation

Yuqian Wang, Renqi Huang, Yougong Lu, Mingqi Liu, Ran Mo*

State Key Laboratory of Natural Medicines, Jiangsu Key Laboratory of Drug Discovery for Metabolic Diseases, Center of Advanced Pharmaceuticals and Biomaterials, School of Life Science and Technology, China Pharmaceutical University, Nanjing 211198, China

*Email: rmo@cpu.edu.cn

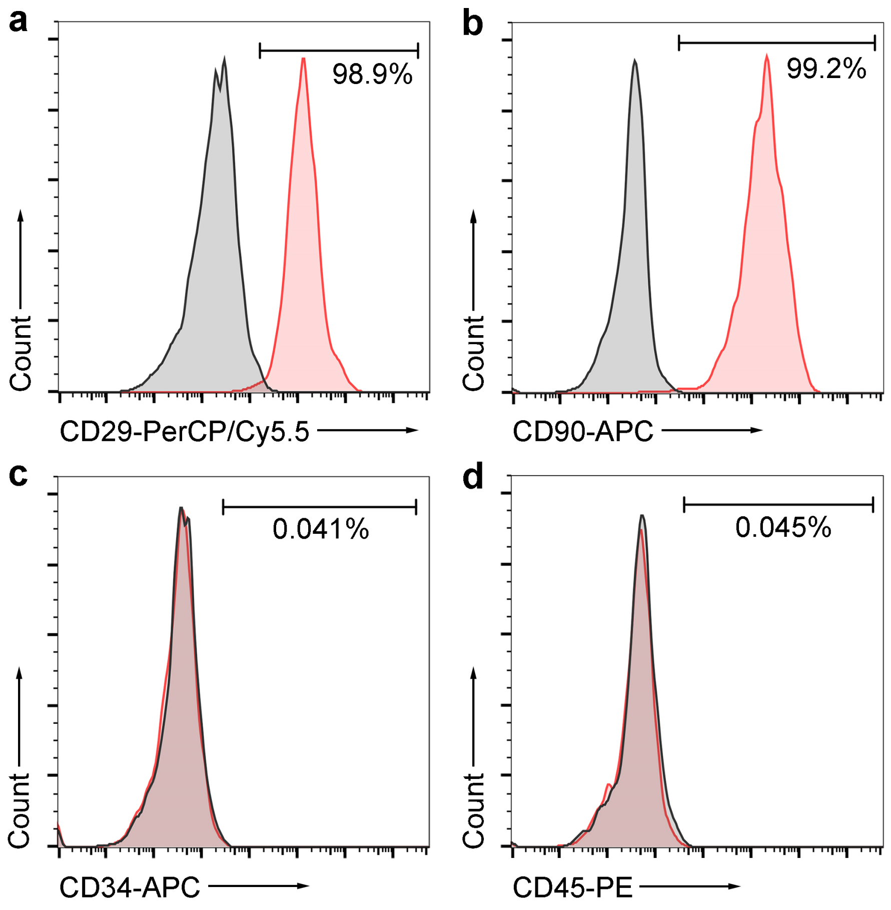

Supplementary Figure 1. Expression of CD29 (**a**), CD90 (**b**), CD34 (**c**) and CD45 (**d**) on MSCs determined by flow cytometry. Representative is displayed from 3 independent experiments.

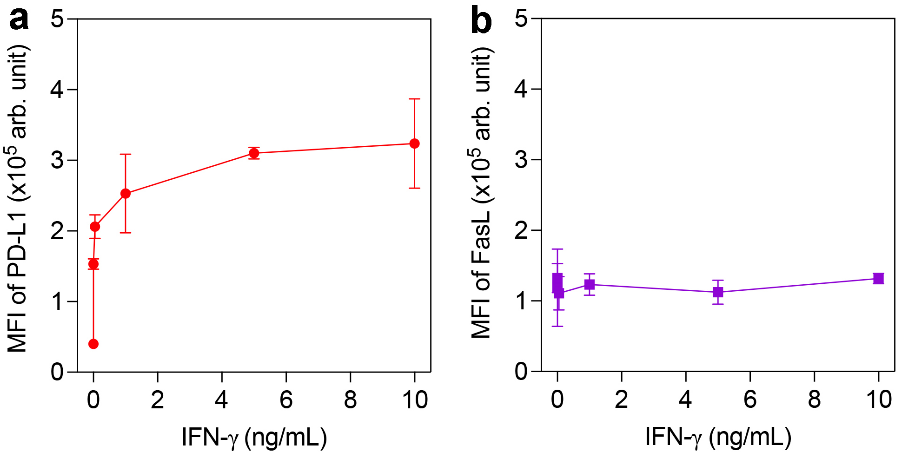

Supplementary Figure 2. Expression of PD-L1 (**a**) and FasL (**b**) on MSCs after treatment with varying concentrations of IFN-γ determined by flow cytometry. MFI, mean fluorescent intensity. arb. unit, arbitrary unit. Data are shown as mean ± s.d. (*n* = 3 independent samples).

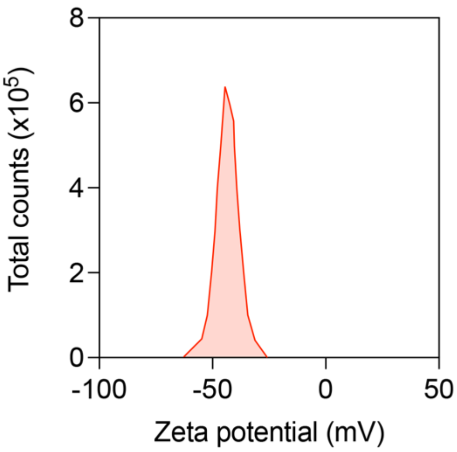

Supplementary Figure 3. Zeta potential of MMVs. Representative is displayed from 3 independent experiments.

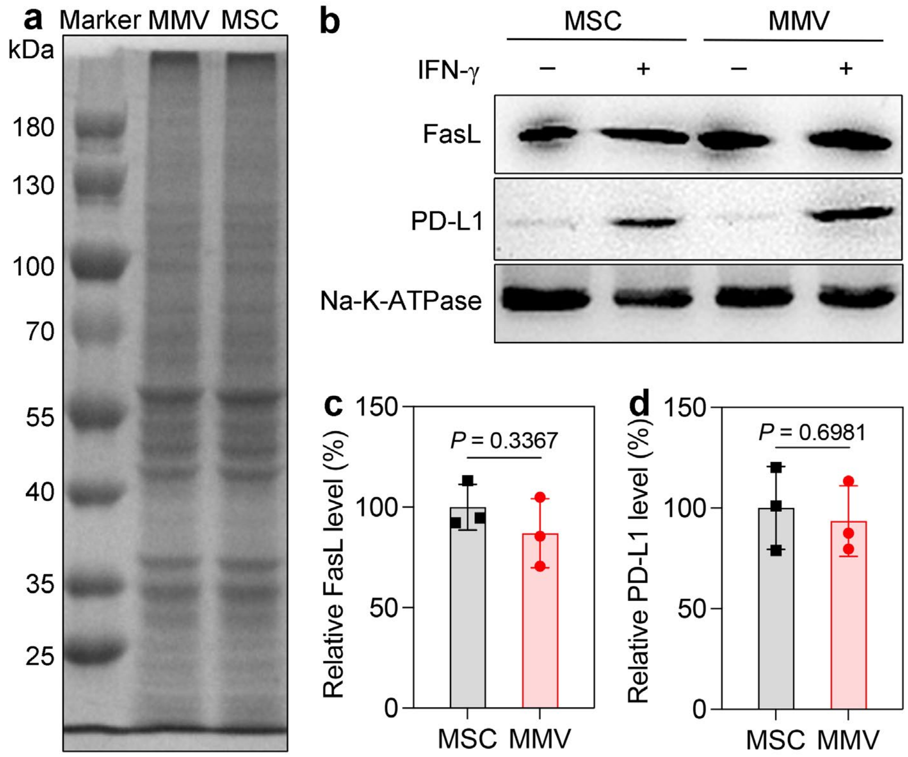

Supplementary Figure 4. **a**, Expression of proteins on MSCs and MMVs examined by SDS-PAGE. **b**, Expression of FasL and PD-L1 on MSCs and MMVs before and after treatment with IFN-γ examined by western blotting. **c**,**d**, Relative expression of FasL (**c**) and PD-L1 (**d**) on the IFN-γ-treated MSCs and MMVs determined by ELISA assay. Representative is displayed from 3 independent experiments (**a**,**b**). Data are shown as mean ± s.d. (n = 3 independent samples in **c**,**d**). Two-tailed unpaired t-test was used for statistical analysis of **c**,**d**.

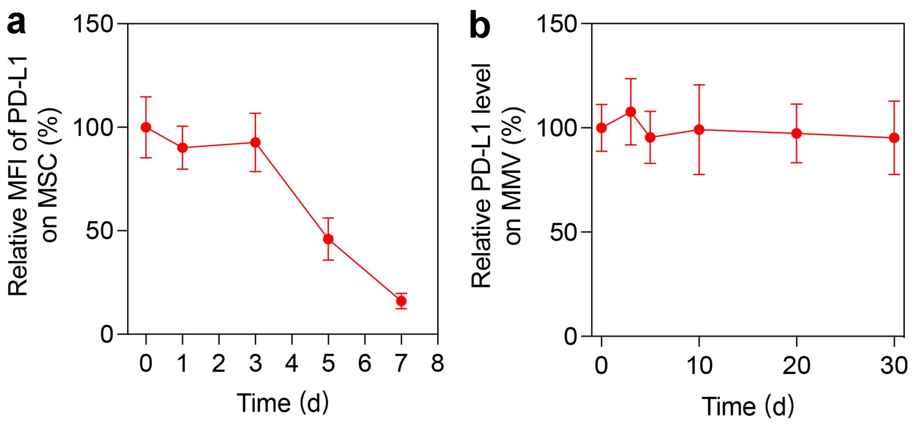

Supplementary Figure 5. **a**, Relative expression of PD-L1 on MSCs within 7 d after removal of IFN-γ determined by flow cytometry. **b**, Relative expression of PD-L1 on MMVs obtained from the IFN-γ-treated MSCs within 30 d quantified by ELISA assay. Data are shown as mean ± s.d. (*n* = 6 independent samples).

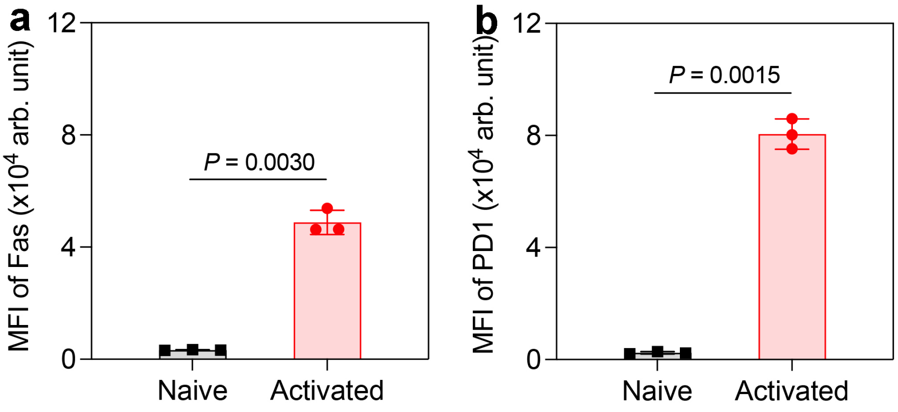

Supplementary Figure 6. Expression of Fas (**a**) and PD1 (**b**) on T cells within the naive and activated splenocytes determined by flow cytometry. Data are shown as mean ± s.d. (n = 3 independent samples). Two-tailed unpaired t-test was used for statistical analysis.

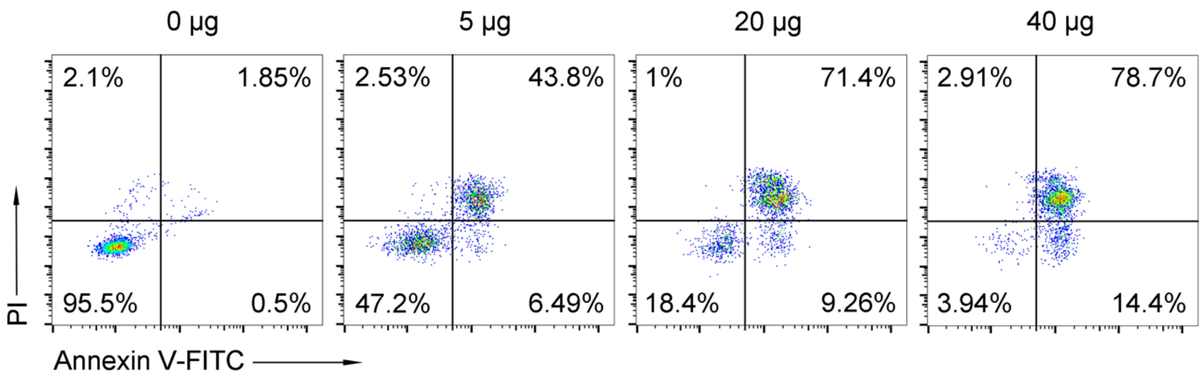

Supplementary Figure 7. Apoptosis of T cells within the activated splenocytes after treatment with varying amounts of MMVs determined by Annexin V-FITC/PI double-staining assay. Representative is displayed from 6 independent experiments.

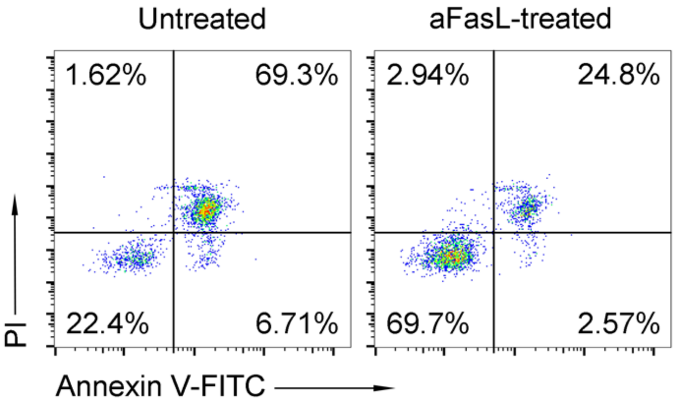

Supplementary Figure 8. Apoptosis of T cells within the activated splenocytes after treatment with MMVs in the absence and presence of aFasL determined by Annexin V-FITC/PI double-staining assay. Representative is displayed from 6 independent experiments.

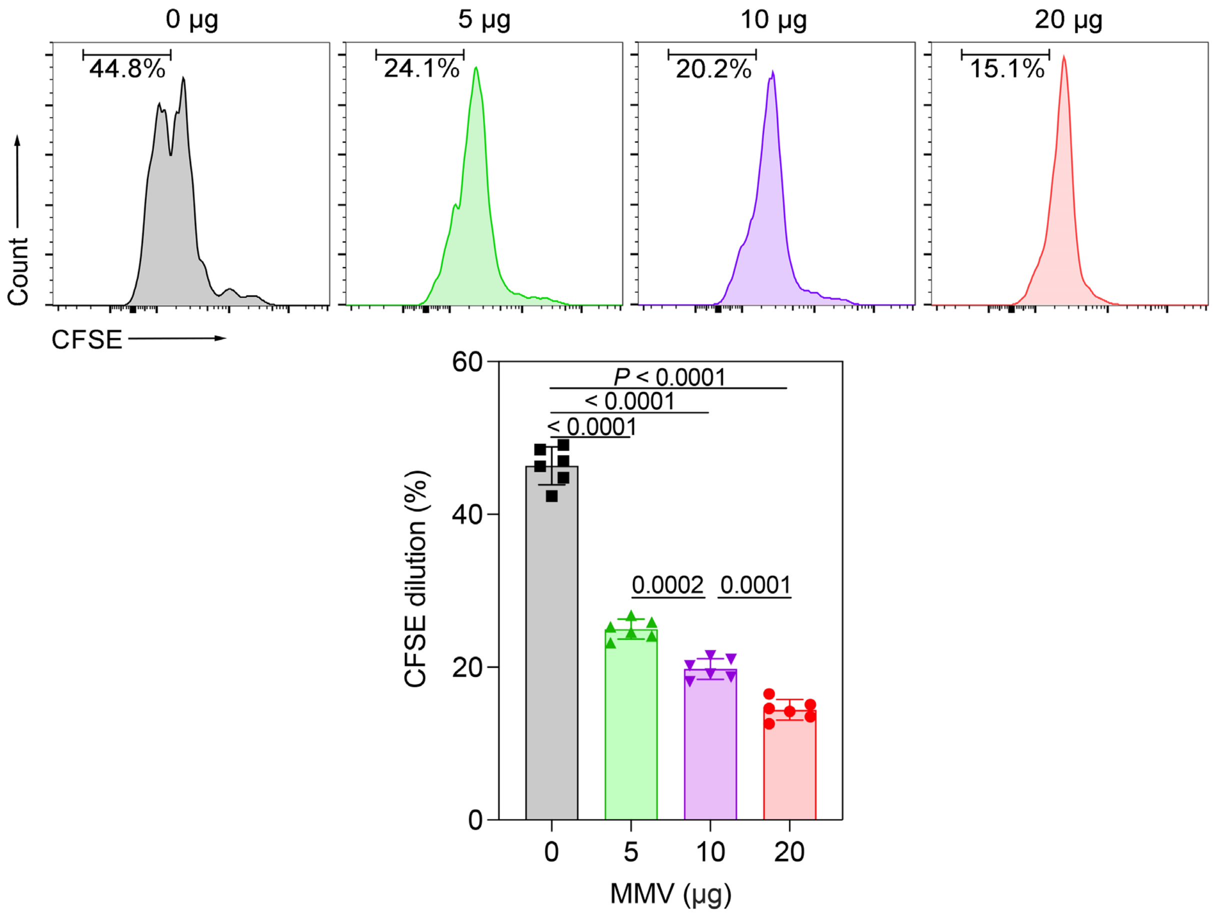

Supplementary Figure 9. Proliferation of T cells within the activated splenocytes after treatment with varying amounts of MMVs determined by CFSE dilution assay. Representative is displayed from 6 independent experiments. Data are shown as mean ± s.d. (n = 6 independent samples). One-way ANOVA with Tukey post-hoc test was used for statistical analysis.

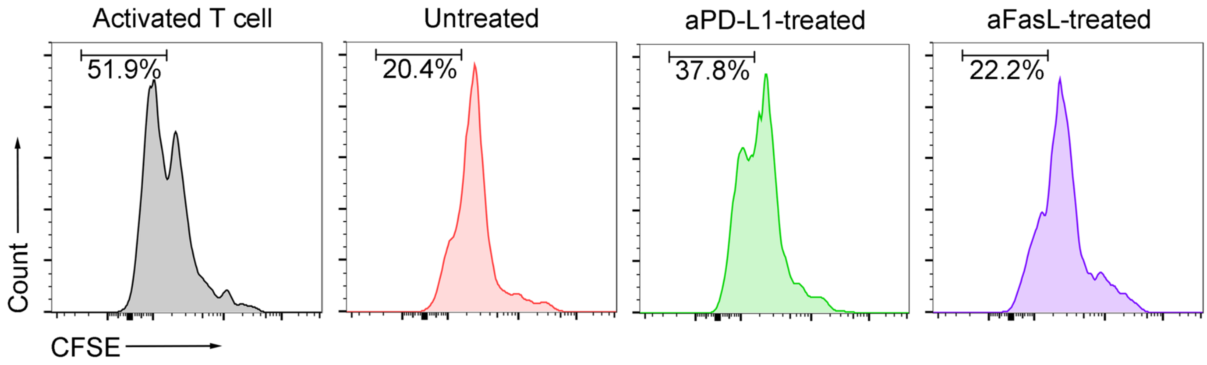

Supplementary Figure 10. Proliferation of T cells within the activated splenocytes after treatment with MMVs in the absence and presence of aPD-L1 or aFasL determined by CFSE dilution assay. Representative is displayed from 6 independent experiments.

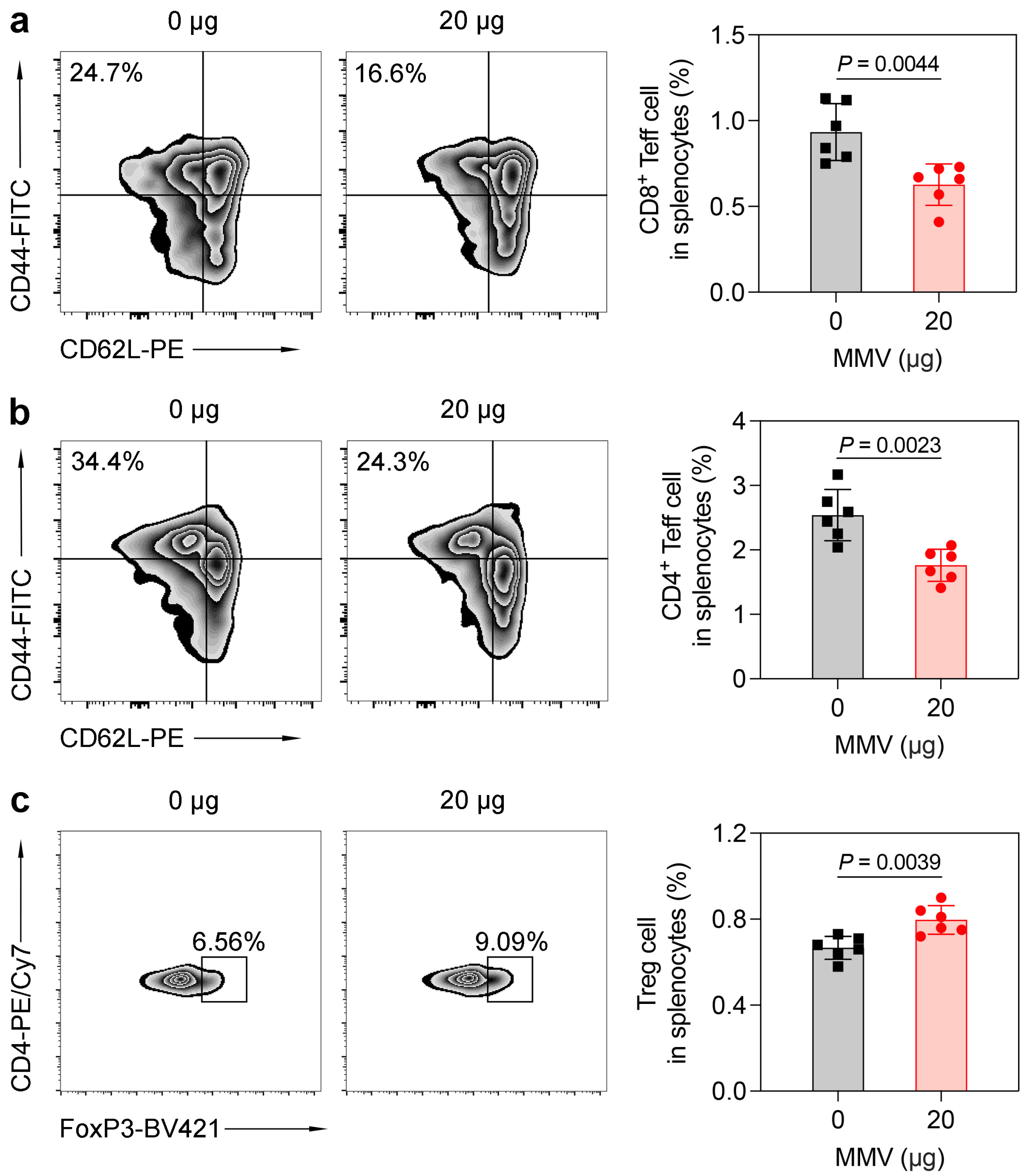

Supplementary Figure 11. Proportion of CD8$^+$ Teff (CD8$^+$CD44$^+$CD62L$^-$) (**a**), CD4$^+$ Teff (CD4$^+$CD44$^+$CD62L$^-$) (**b**) and Treg (CD4$^+$FoxP3$^+$) (**c**) cells in the activated splenocytes after treatment with MMVs determined by flow cytometry. The flow cytometric plots present the percentages of CD44$^+$CD62L$^-$ cell populations in the CD8$^+$ (**a**) and CD4$^+$ (**b**) T cells and FoxP3$^+$ cell populations in the CD4$^+$ T cells (**c**). Representative is displayed from 6 independent experiments. Data are shown as mean ± s.d. (n = 6 independent samples). Two-tailed unpaired t-test was used for statistical analysis.

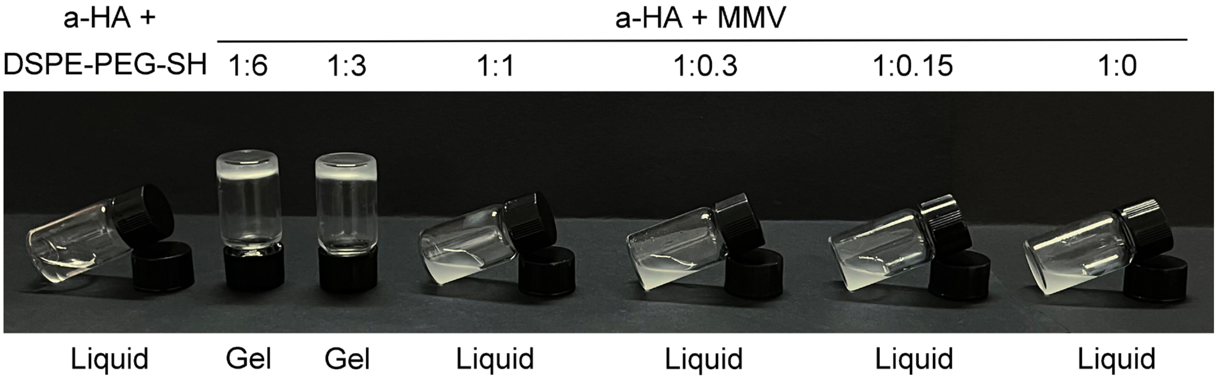

Supplementary Figure 12. Gelation of the mixture of a-HA and the DSPE-PEG-SH-anchored MMVs with varying modification ratios (MMV:DSPE-PEG-SH, mg protein:mg) examined by tube inversion assay. Representative is displayed from 3 independent experiments.

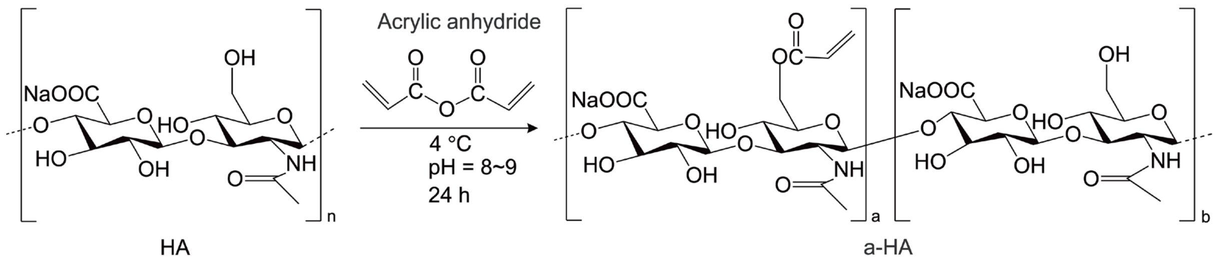

Supplementary Figure 13. Synthetic route of a-HA.

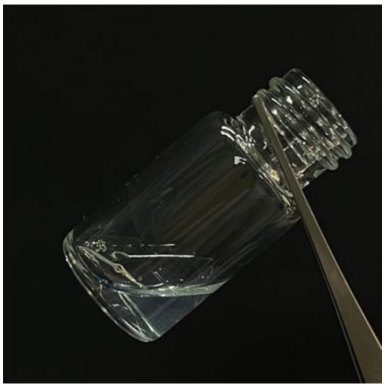

Supplementary Figure 14. Image of MMV-Gel after treatment with Triton X-100 (10%). Representative is displayed from 3 independent experiments.

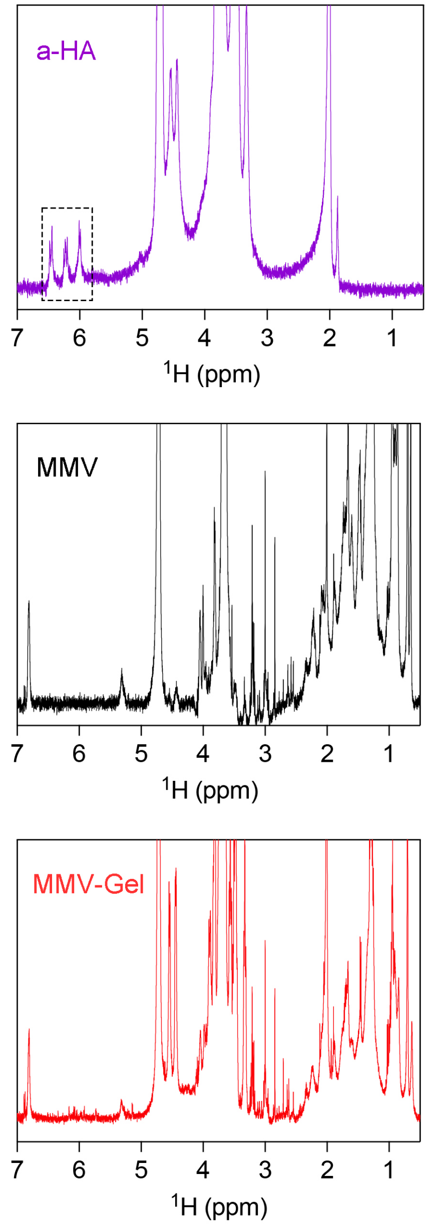

Supplementary Figure 15. ^{1}H NMR spectra of a-HA, MMV and MMV-Gel. Representative is displayed from 3 independent experiments. Black dotted box indicates the acrylic acid proton peaks.

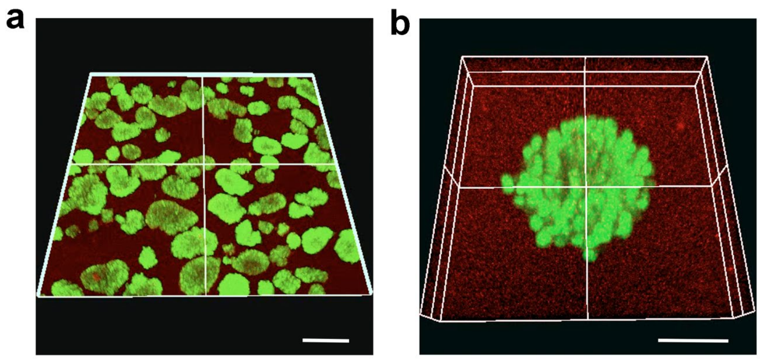

Supplementary Figure 16. Three-dimensional reconstructed confocal microscopic images of islet/Rho-MMV-Gel stained with acridine orange at low (**a**) and high (**b**) magnification. Representative is displayed from 3 independent experiments. Scale bars, 200 μm (**a**) and 50 μm (**b**).

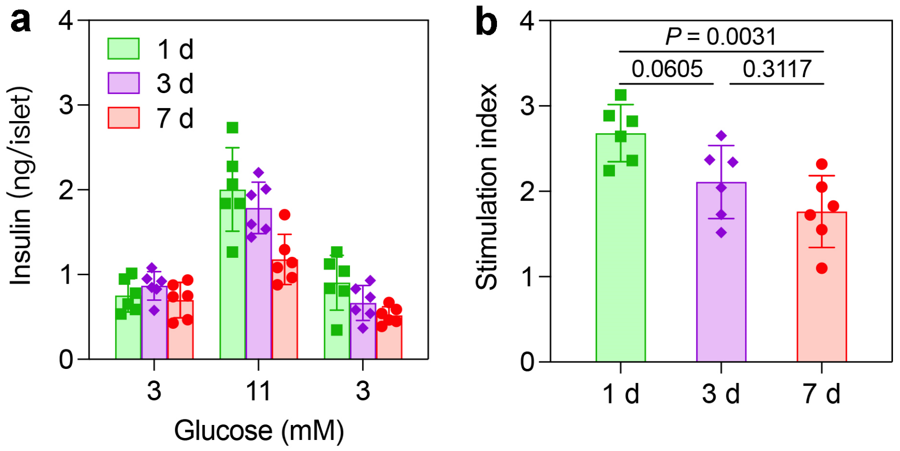

Supplementary Figure 17. GSIS (**a**) and stimulation index (**b**) of free islet within 7 d. Data are shown as mean ± s.d. (n = 6 independent samples). One-way ANOVA with Tukey post-hoc test was used for statistical analysis of **b**.

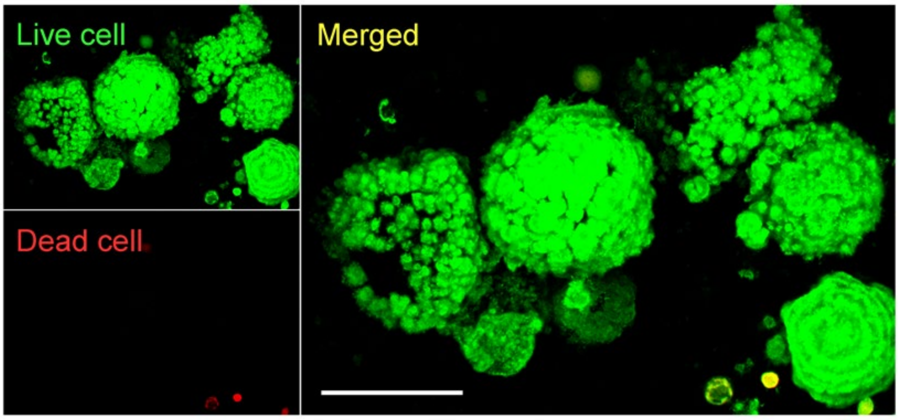

Supplementary Figure 18. Fluorescent imaging of viability of islet/MMV-Gel for 24 h examined by acridine orange/PI double-staining assay. Representative is displayed from 3 independent experiments. Scale bar, 50 μm.

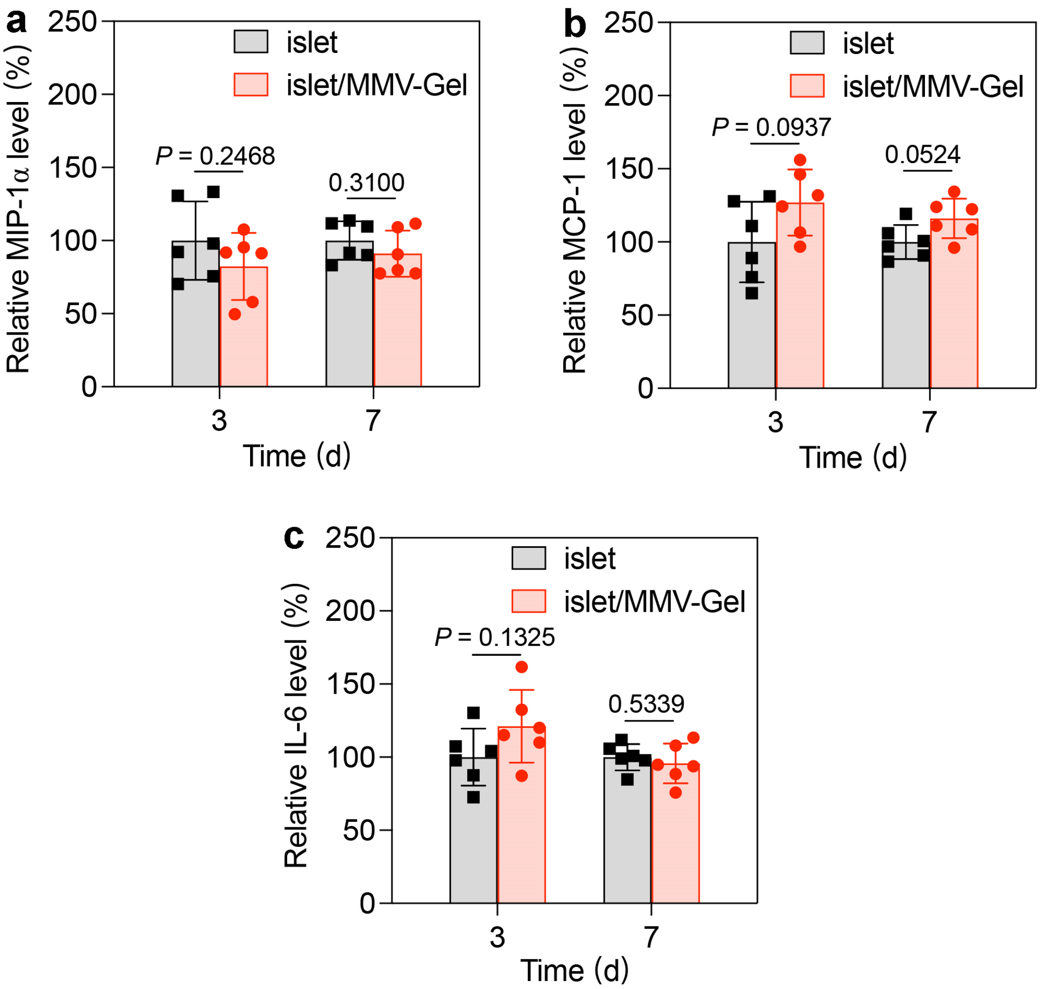

Supplementary Figure 19. Secretion of MIP-1α (**a**), MCP-1 (**b**) and IL-6 (**c**) from islet/MMV-Gel relative to free islet within 7 d. MIP-1α, macrophage inflammatory protein-1α. MCP-1, monocyte chemoattractant protein-1. IL-6, interleukin-6. Data are shown as mean ± s.d. ($n = 6$ independent samples). Two-tailed unpaired t-test was used for statistical analysis.

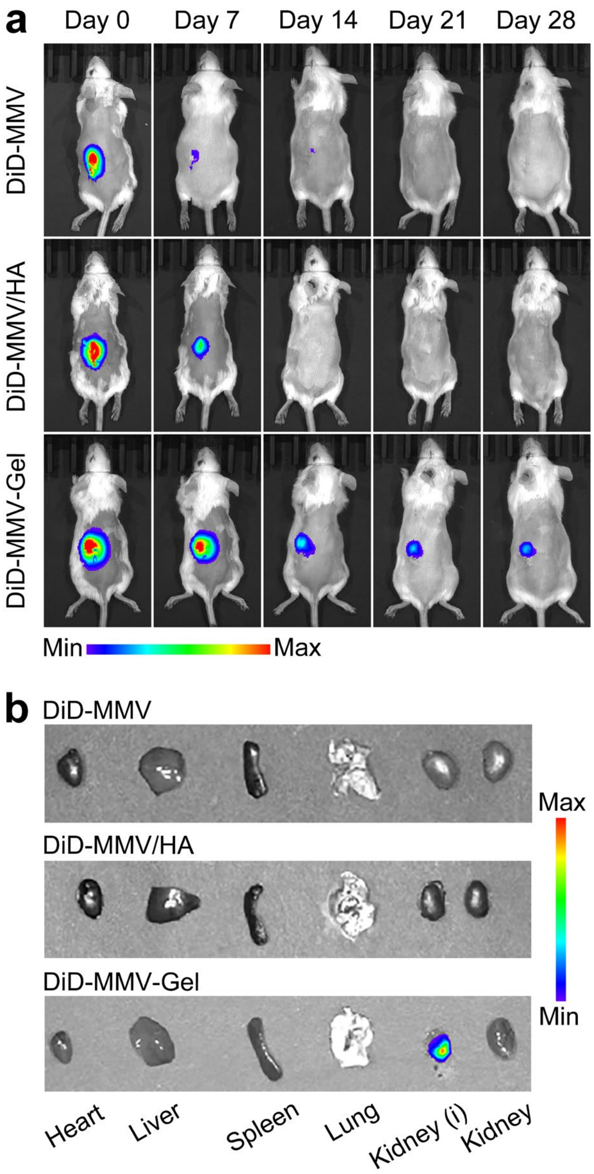

Supplementary Figure 20. a, Fluorescent images of the mice after implantation of DiD-MMV, DiD-MMV/HA (the mixture of DiD-MMV and HA) and DiD-MMV-Gel under the kidney capsule within 28 d. Experiments were repeated three times independently. **b**, Fluorescent images of five major tissues harvested from the normal mice at 28 d post-implantation. Kidney (i), kidney with implantation. Representative is displayed from 3 independent experiments.

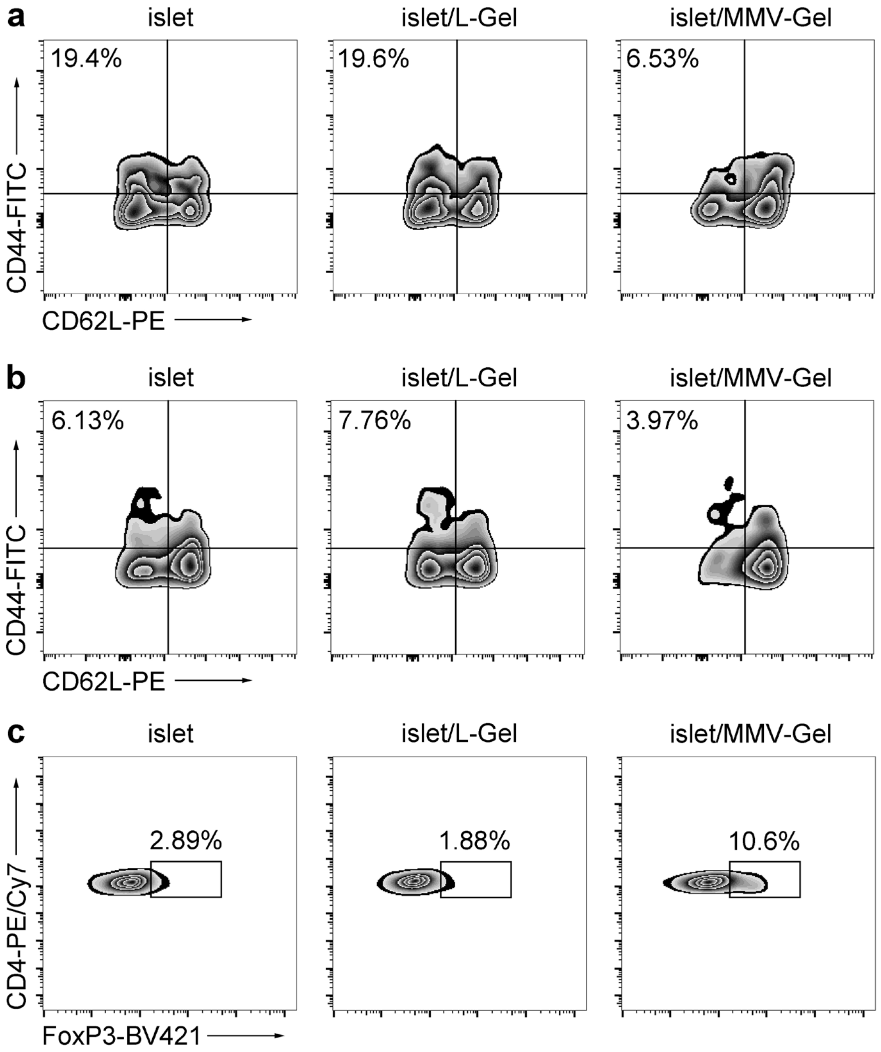

Supplementary Figure 21. Percentages of CD44^{+}CD62L^{-} cell populations in the CD8^{+} (**a**) and CD4^{+} (**b**) T cells and FoxP3^{+} cell populations in the CD4^{+} T cells (**c**) in the transplanted kidney at 7 d post-transplantation of islet, islet/L-Gel and islet/MMV-Gel determined by flow cytometry. Representative is displayed from 6 independent experiments.

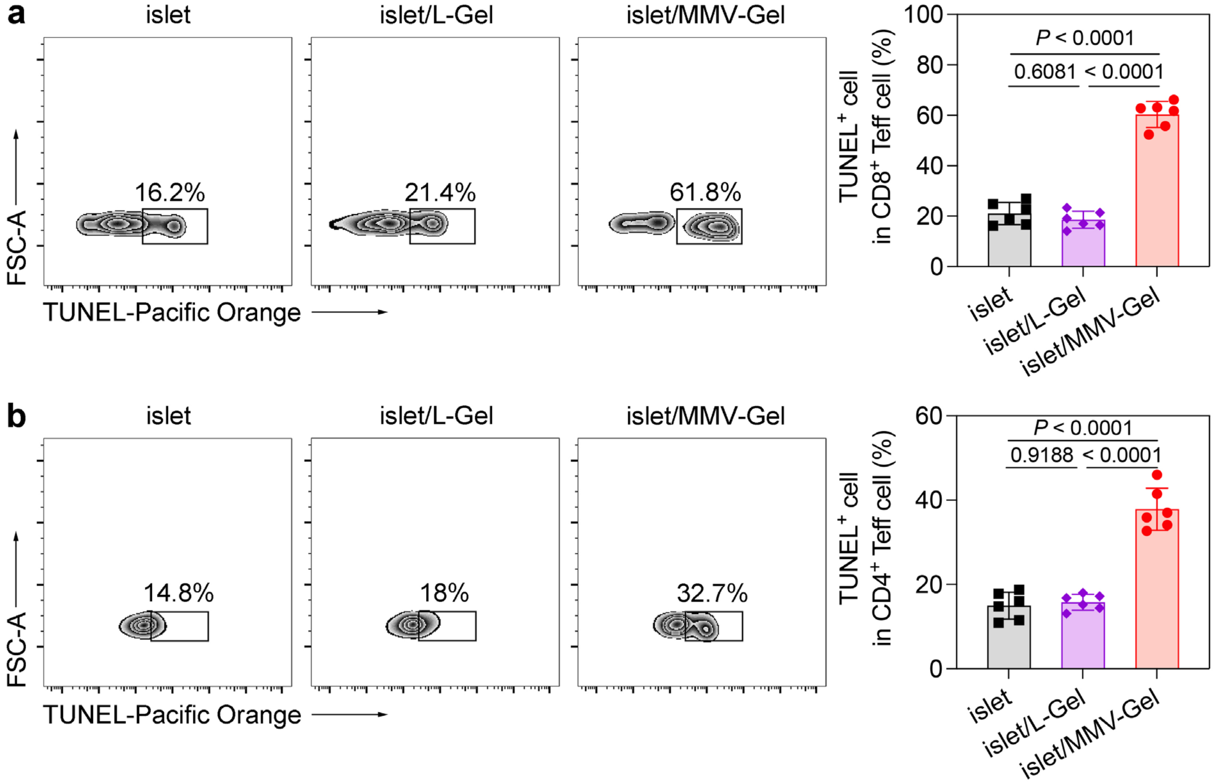

Supplementary Figure 22. Percentages of TUNEL$^+$ cells in the CD8$^+$ Teff (**a**) and CD4$^+$ Teff (**b**) cells in the transplanted kidney at 7 d post-transplantation of islet, islet/L-Gel and islet/MMV-Gel determined by flow cytometry. Representative is displayed from 6 independent experiments. Data are shown as mean ± s.d. (n = 6 mice). One-way ANOVA with Tukey post-hoc test was used for statistical analysis.

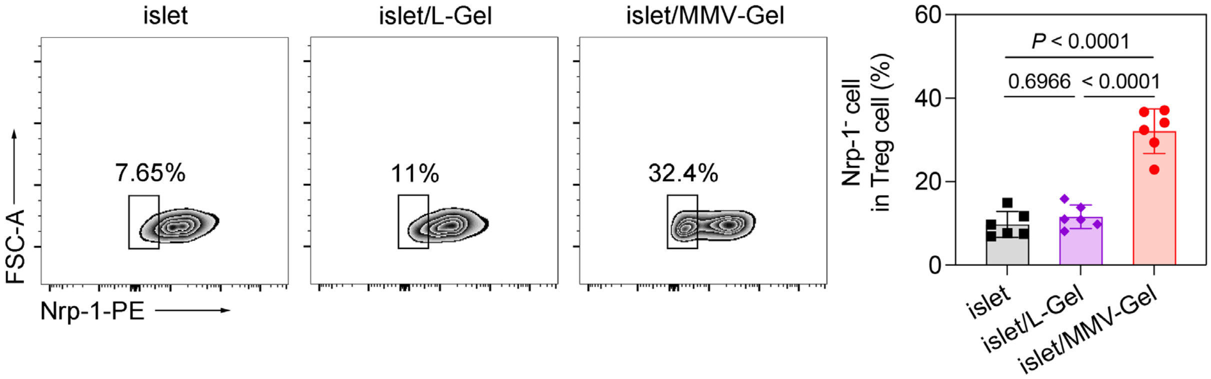

Supplementary Figure 23. Percentages of iTreg (CD4$^+$FoxP3$^+$Nrp-1$^-$) cells in the Treg cells in the transplanted kidney at 7 d post-transplantation of islet, islet/L-Gel and islet/MMV-Gel determined by flow cytometry. Representative is displayed from 6 independent experiments. Data are shown as mean ± s.d. (n = 6 mice). One-way ANOVA with Tukey post-hoc test was used for statistical analysis.

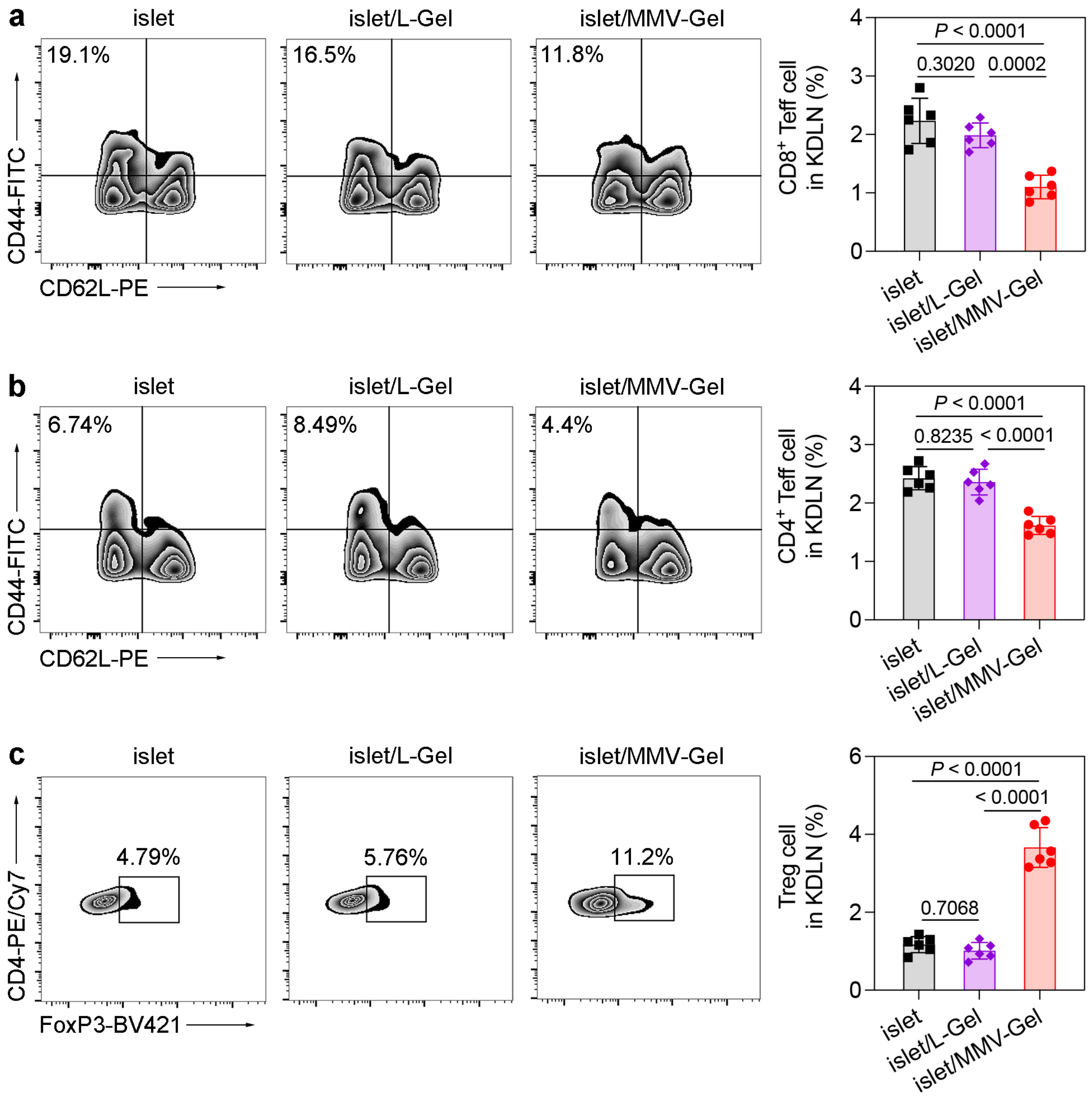

Supplementary Figure 24. Proportions of CD8$^+$ Teff (CD8$^+$CD44$^+$CD62L$^-$) (**a**), CD4$^+$ Teff (CD4$^+$CD44$^+$CD62L$^-$) (**b**) and Treg (CD4$^+$FoxP3$^+$) (**c**) cells in KDLN at 7 d post-transplantation of islet, islet/L-Gel and islet/MMV-Gel determined by flow cytometry. The flow cytometric plots present the percentages of CD44$^+$CD62L$^-$ cell populations in the CD8$^+$ (**a**) and CD4$^+$ (**b**) T cells and FoxP3$^+$ cell populations in the CD4$^+$ T cells (**c**). Representative is displayed from 6 independent experiments. Data are shown as mean ± s.d. (n = 6 mice). One-way ANOVA with Tukey post-hoc test was used for statistical analysis.

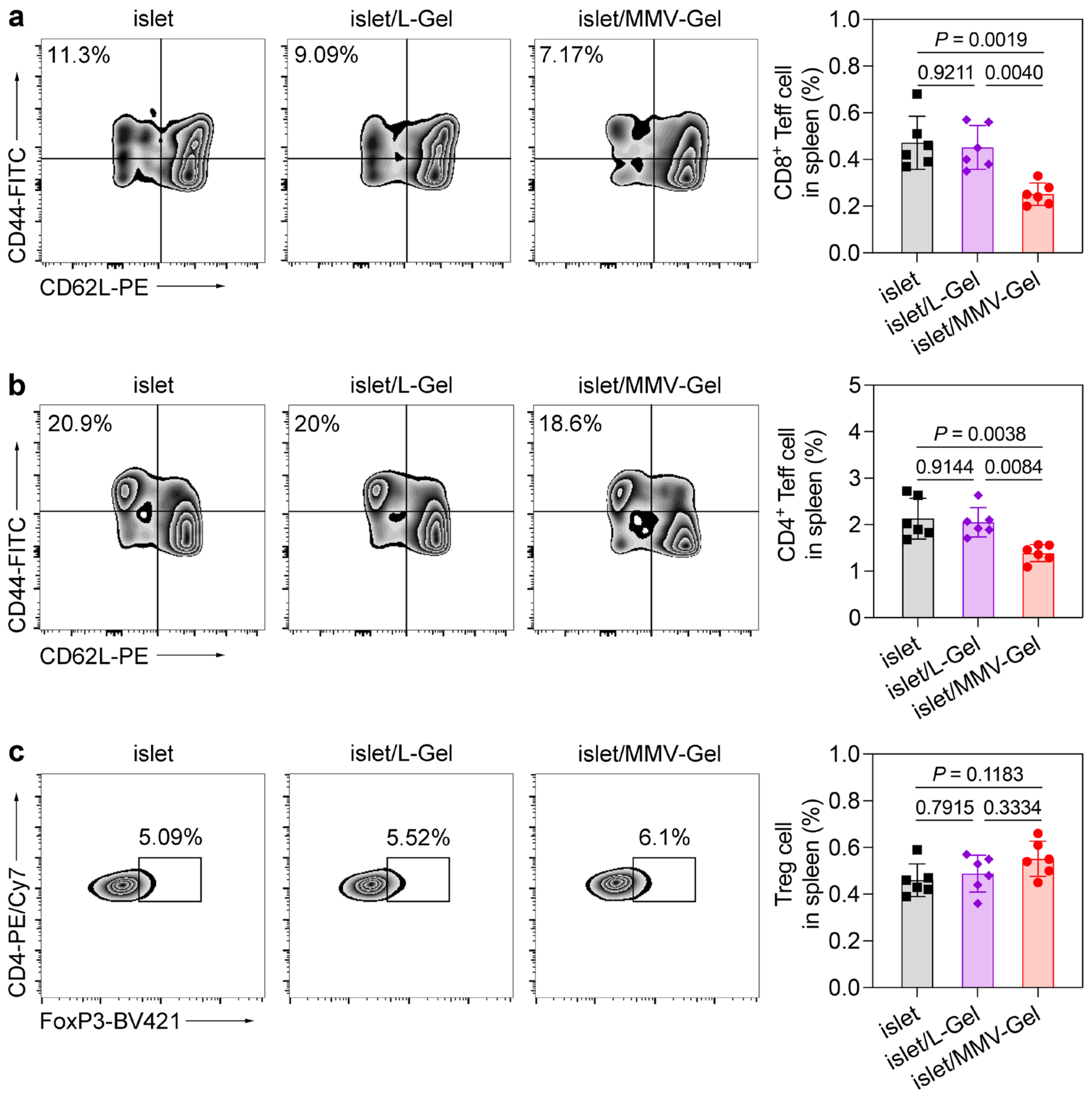

Supplementary Figure 25. Proportions of $CD8^+$ Teff ($CD8^+CD44^+CD62L^-$) (**a**), $CD4^+$ Teff ($CD4^+CD44^+CD62L^-$) (**b**) and Treg ($CD4^+FoxP3^+$) (**c**) cells in spleen at 7 d post-transplantation of islet, islet/L-Gel and islet/MMV-Gel determined by flow cytometry. The flow cytometric plots present the percentages of $CD44^+CD62L^-$ cell populations in the $CD8^+$ (**a**) and $CD4^+$ (**b**) T cells and $FoxP3^+$ cell populations in the $CD4^+$ T cells (**c**). Representative is displayed from 6 independent experiments. Data are shown as mean ± s.d. (n = 6 mice). One-way ANOVA with Tukey post-hoc test was used for statistical analysis.

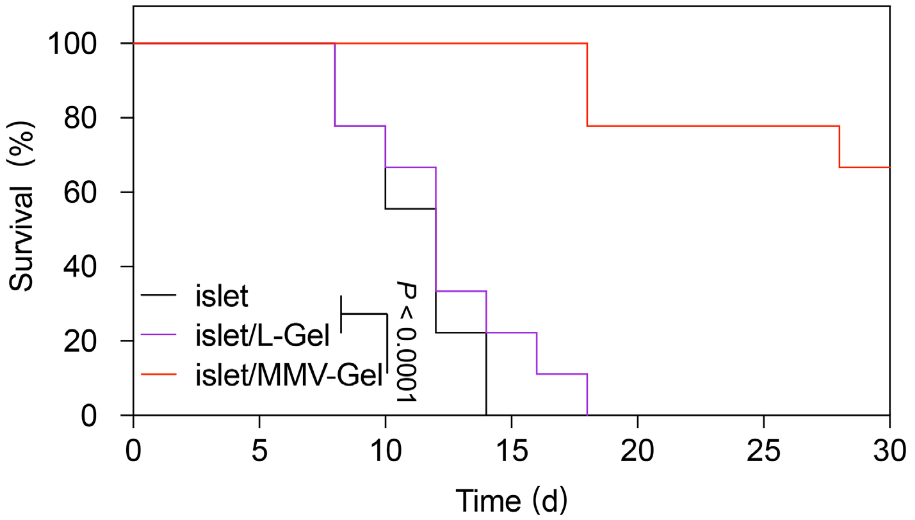

Supplementary Figure 26. Survival of islet, islet/L-Gel and islet/MMV-Gel ($n = 9$ mice). Two-sided log-rank (Mantel–Cox) test was used for statistical analysis.

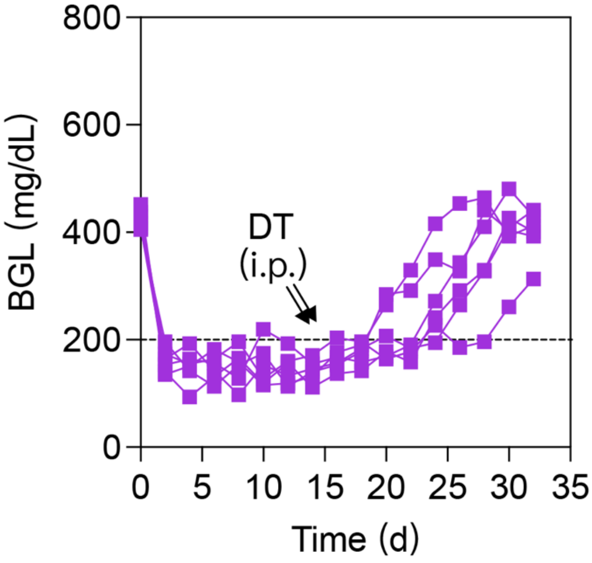

Supplementary Figure 27. Changes in the individual BGLs of the islet/MMV-Gel-transplanted FoxP3/DTR diabetic mice after i.p. injection of DT (n = 6 mice). Black arrows indicate two i.p. injections of DT at Day 14 and 15.

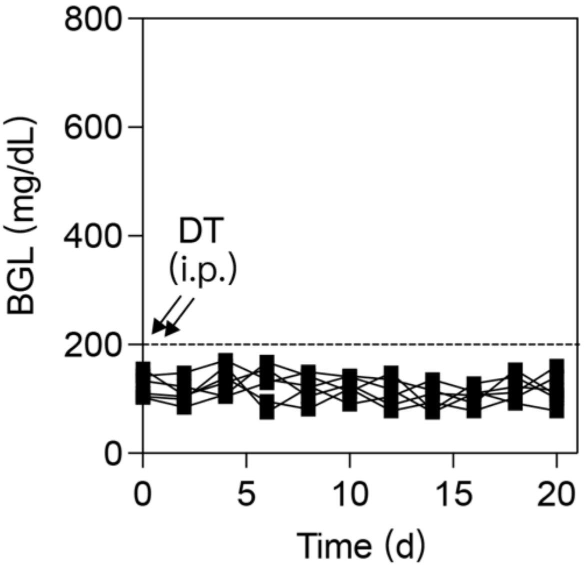

Supplementary Figure 28. Changes in the individual BGLs of the FoxP3/DTR mice after i.p. injection of DT (n = 6 mice). Black arrows indicate two i.p. injections of DT at Day 0 and 1.

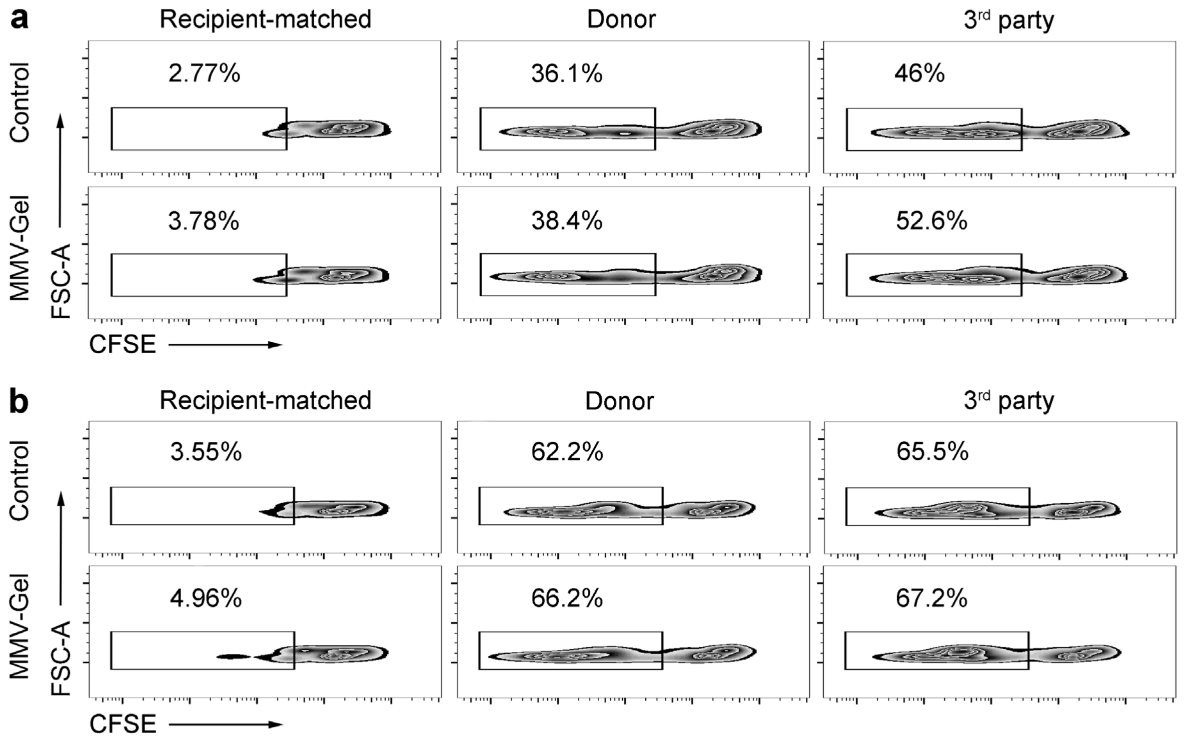

Supplementary Figure 29. Proliferative responses of CD8$^+$ (**a**) and CD4$^+$ (**b**) T cells in spleen from the BALB/c mice at 30 d post-transplantation of MMV-Gel to the splenocytes isolated from the BALB/c (recipient-matched), C57BL/6 (donor) and C3H (3rd party) mice determined by flow cytometry. Representative is displayed from 6 independent experiments.

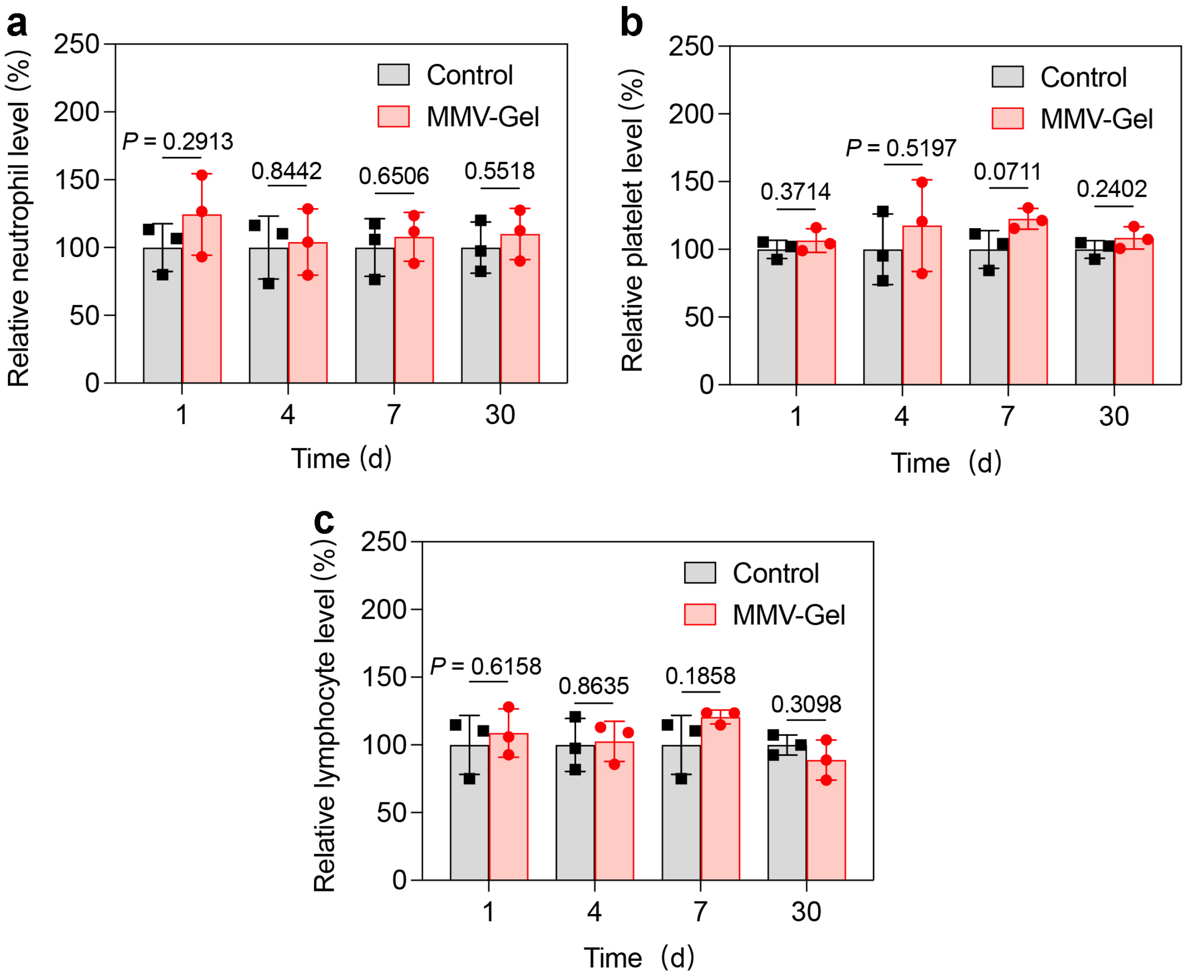

Supplementary Figure 30. Relative levels of neutrophil (**a**), platelet (**b**) and lymphocyte (**c**) in blood of normal over time post-transplantation with MMV-Gel. Data are shown as mean ± s.d. (n = 3 mice). Two-tailed unpaired t-test was used for statistical analysis.

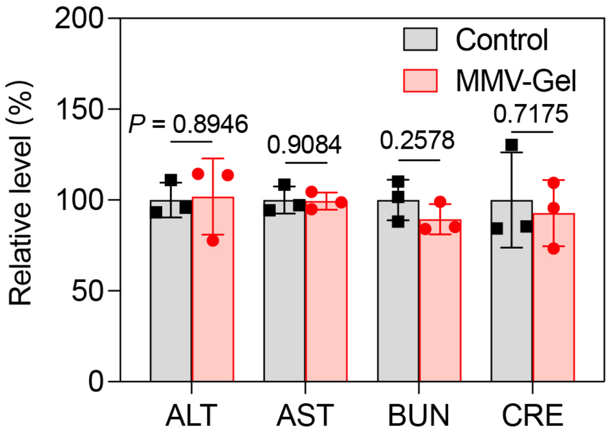

Supplementary Figure 31. Relative serum levels of ALT, AST, BUN and CRE of normal mice at 60 d post-transplantation of MMV-Gel. ALT, alanine aminotransferase. AST, aspartate transaminase. BUN, blood urea nitrogen. CRE, creatinine. Data are shown as mean ± s.d. (n = 3 mice). Two-tailed unpaired t-test was used for statistical analysis.

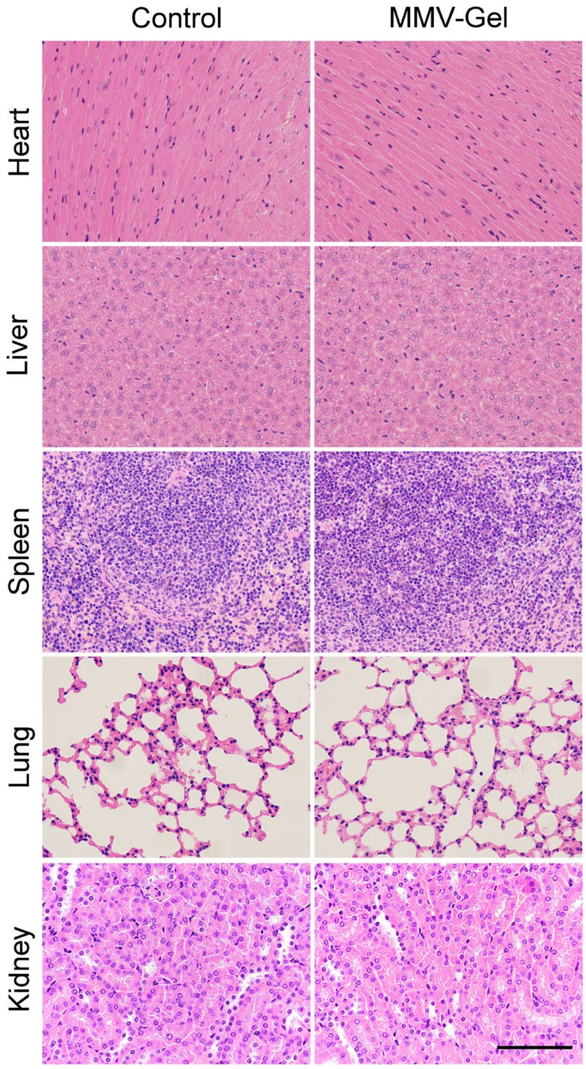

Supplementary Figure 32. Histological examination of five major tissues harvested from normal mice at 60 d post-transplantation of MMV-Gel by H&E staining. Representative is displayed from 3 independent experiments. Scale bar, 100 μm.

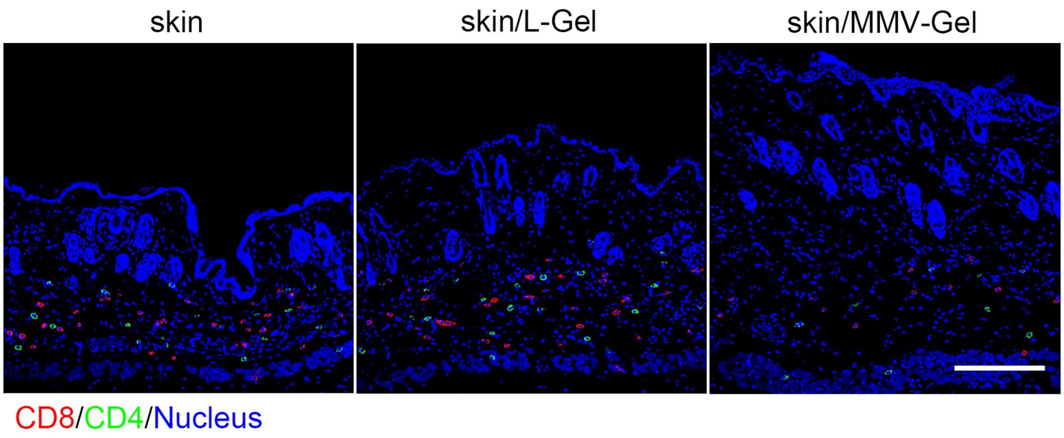

Supplementary Figure 33. Infiltration of $CD8^+$ and $CD4^+$ T cells in the transplanted skin at 7 d post-transplantation of skin, skin/L-Gel and skin/MMV-Gel examined by immunofluorescent staining. Representative is displayed from 3 independent experiments. Scale bar, 200 μm.

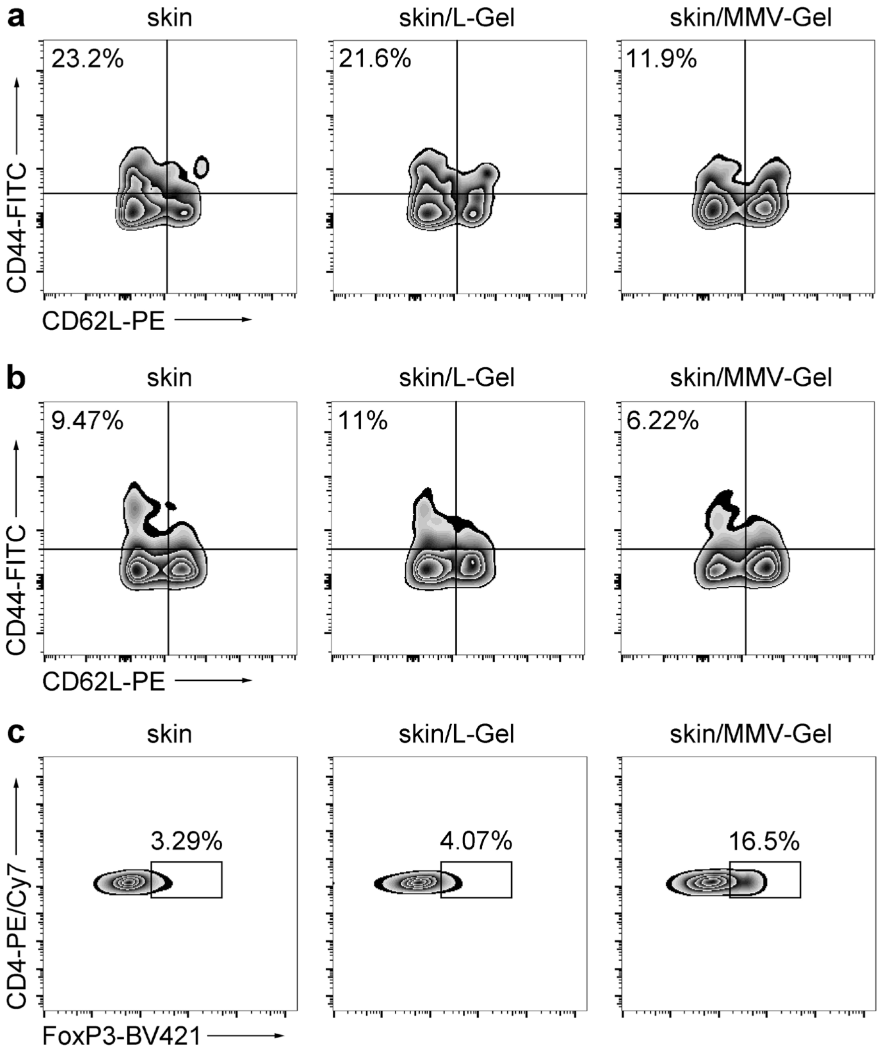

Supplementary Figure 34. Percentages of $CD44^+CD62L^-$ cell populations in the $CD8^+$ (**a**) and $CD4^+$ (**b**) T cells and $FoxP3^+$ cell populations in the $CD4^+$ T cells (**c**) in skin graft at 7 d post-transplantation of skin, skin/L-Gel and skin/MMV-Gel determined by flow cytometry. Representative is displayed from 6 independent experiments.

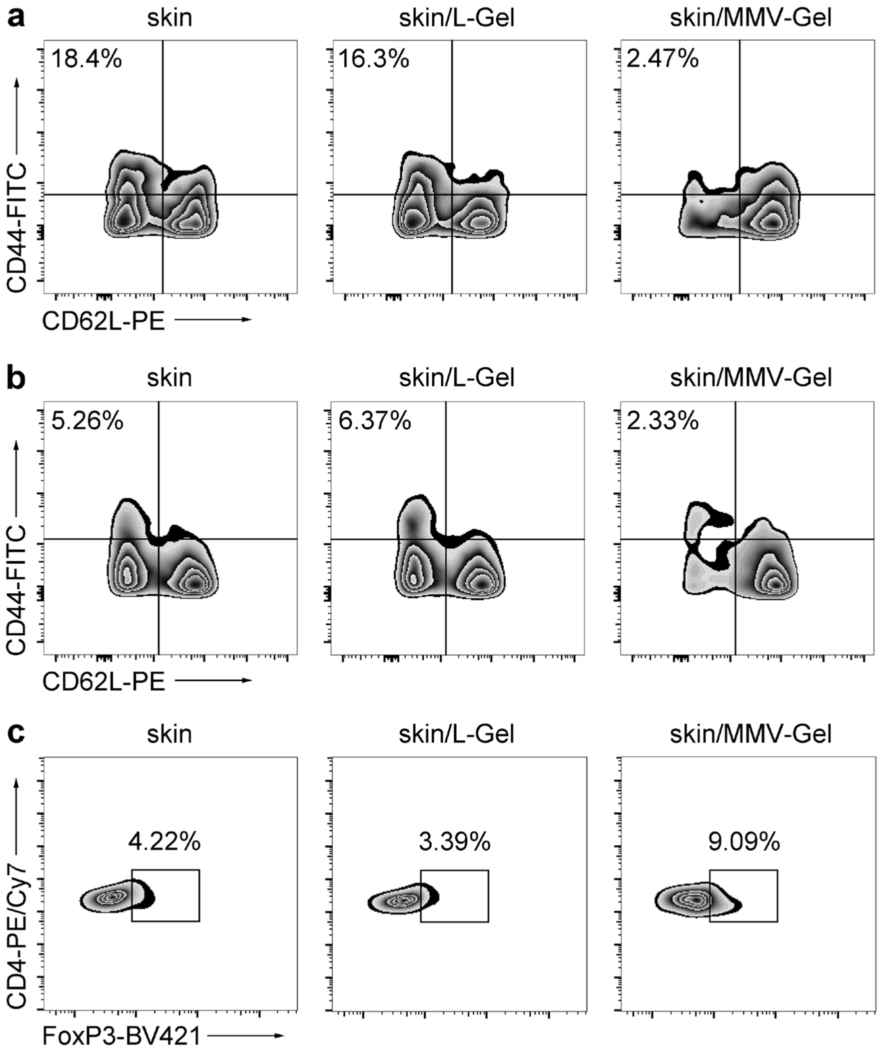

Supplementary Figure 35. Percentages of $CD44^{+}CD62L^{-}$ cell populations in the $CD8^{+}$ (**a**) and $CD4^{+}$ (**b**) T cells and $FoxP3^{+}$ cell populations in the $CD4^{+}$ T cells (**c**) in SDLN at 7 d post-transplantation of skin, skin/L-Gel and skin/MMV-Gel determined by flow cytometry. Representative is displayed from 6 independent experiments.

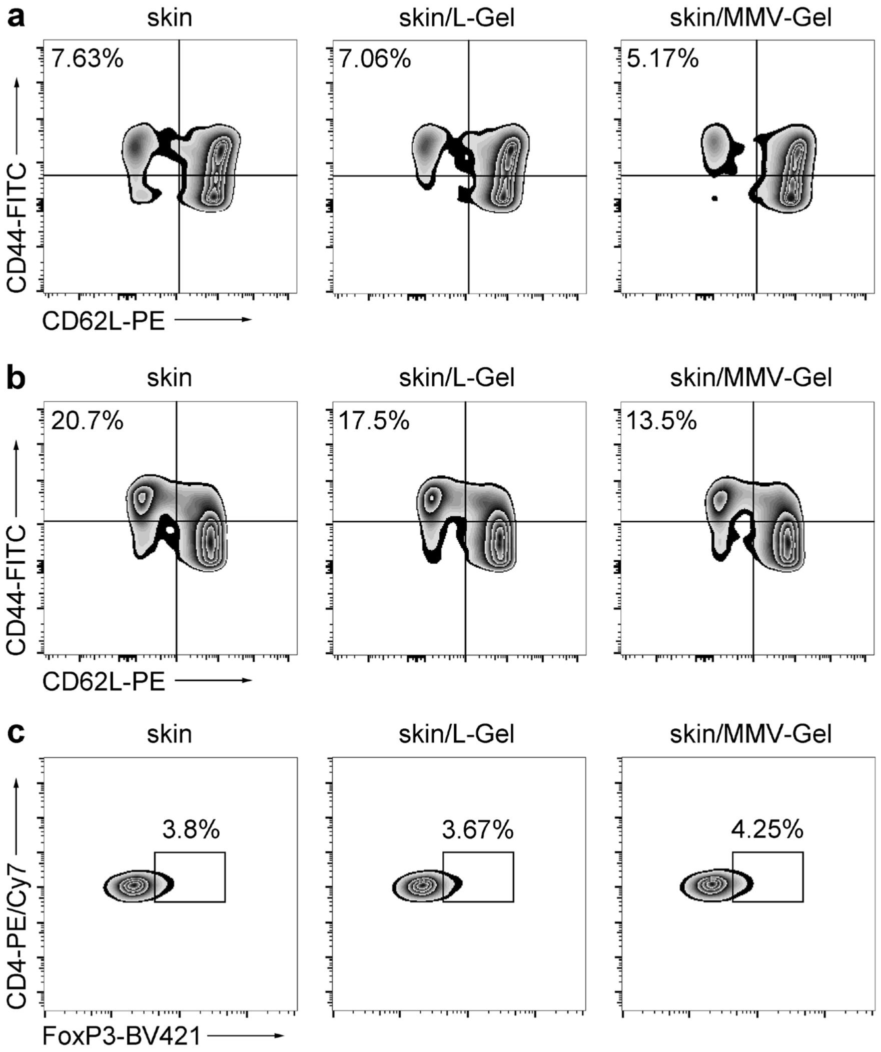

Supplementary Figure 36. Percentages of CD44^{+}CD62L^{-} cell populations in the CD8^{+} (**a**) and CD4^{+} (**b**) T cells and FoxP3^{+} cell populations in the CD4^{+} T cells (**c**) in spleen at 7 d post-transplantation of skin, skin/L-Gel and skin/MMV-Gel determined by flow cytometry. Representative is displayed from 6 independent experiments.

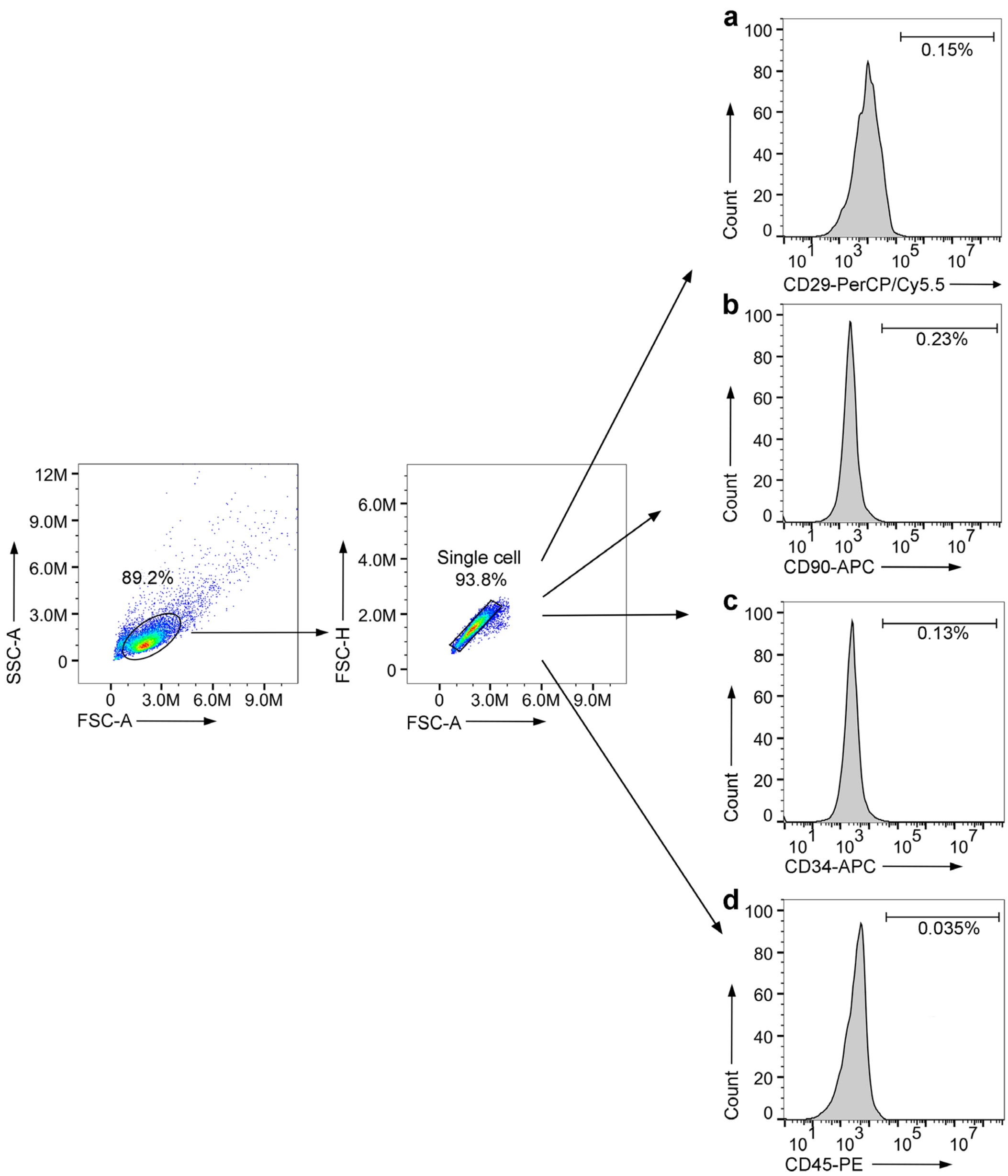

Supplementary Figure 37. Gating strategy for flow cytometric analysis on the expression of CD29 (**a**), CD90 (**b**), CD34 (**c**) and CD45 (**d**) on the surface markers of MSCs (Supplementary Fig. 1).

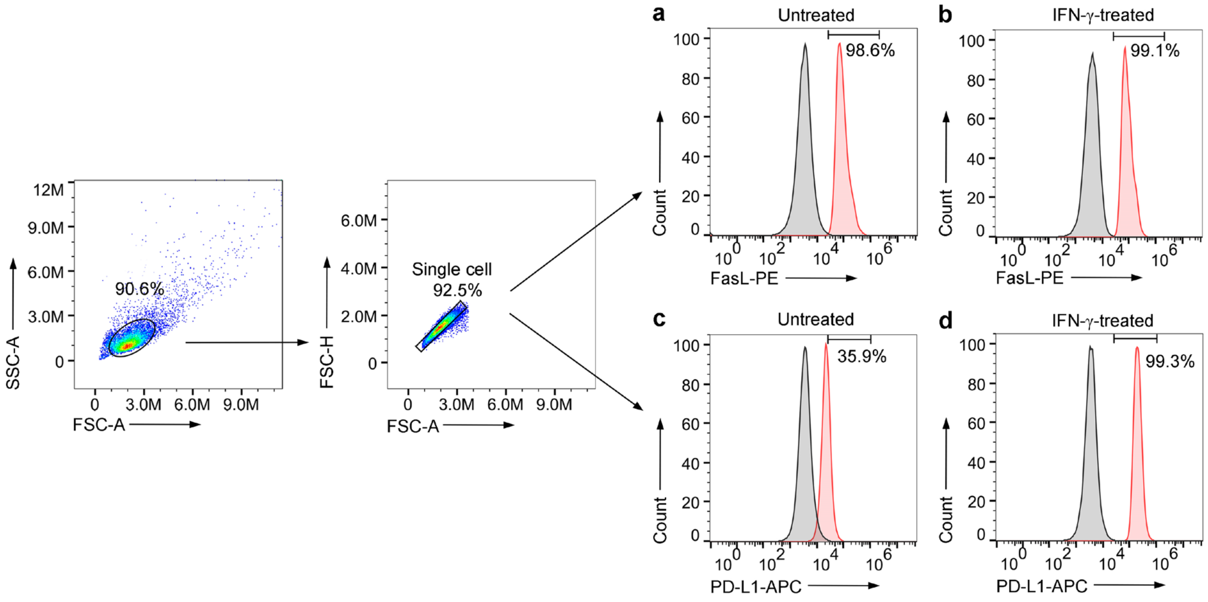

Supplementary Figure 38. Gating strategy for flow cytometric analysis on the expression of FasL (**a,b**) and PD-L1 (**c,d**) on the untreated and IFN-γ-treated MSCs (Fig. 2a, Fig. 2b and Supplementary Fig. 2).

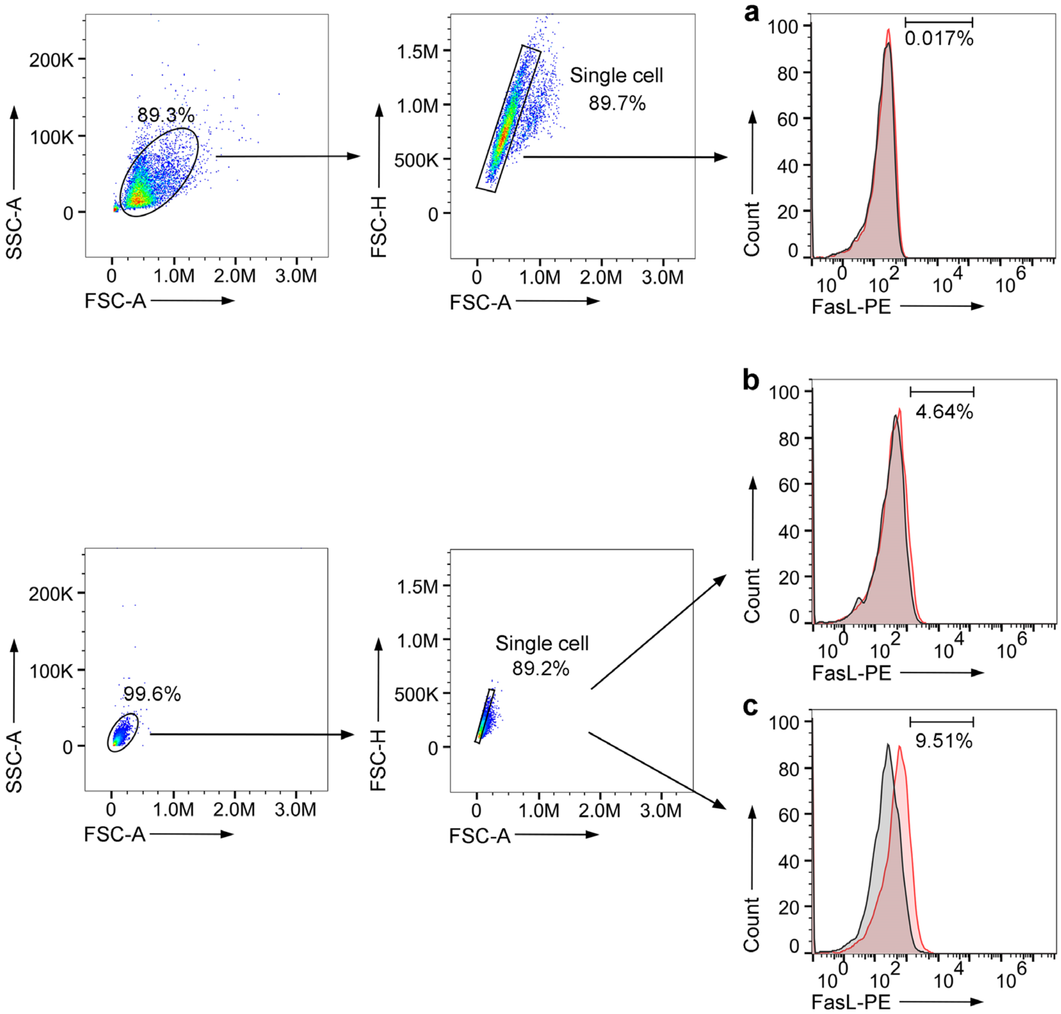

Supplementary Figure 39. Gating strategy for flow cytometric analysis on the expression of FasL on the surface of RBCs (**a**), PLTs (**b**) and aPLTs (**c**) (Fig. 2a).

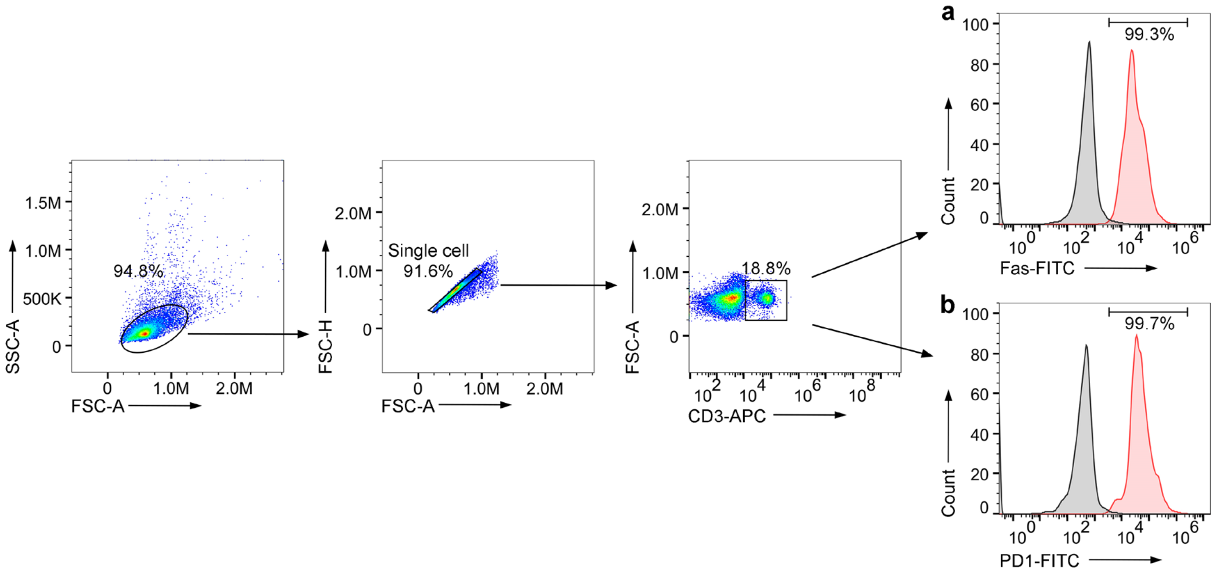

Supplementary Figure 40. Gating strategy for flow cytometric analysis on the expression of Fas (**a**) and PD1 (**b**) on the surface of T cells within the activated splenocytes (Supplementary Fig. 6).

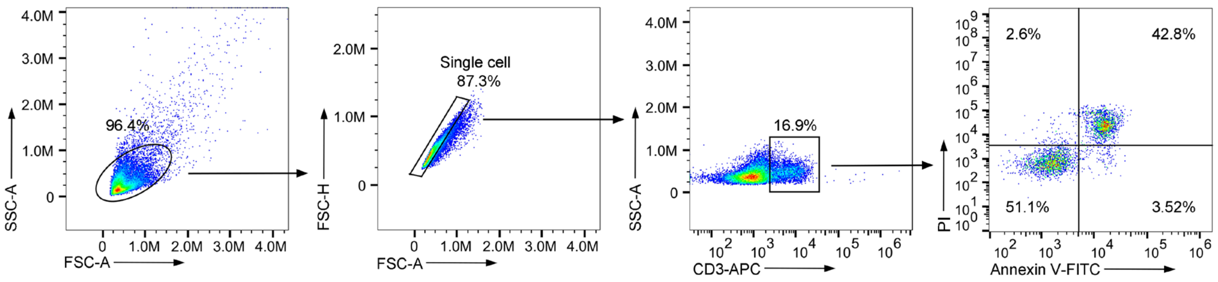

Supplementary Figure 41. Gating strategy for flow cytometric analysis on the proportion of apoptotic T cells within the activated splenocytes (Fig. 2d, Fig. 2e, Supplementary Fig. 7 and Supplementary Fig. 8).

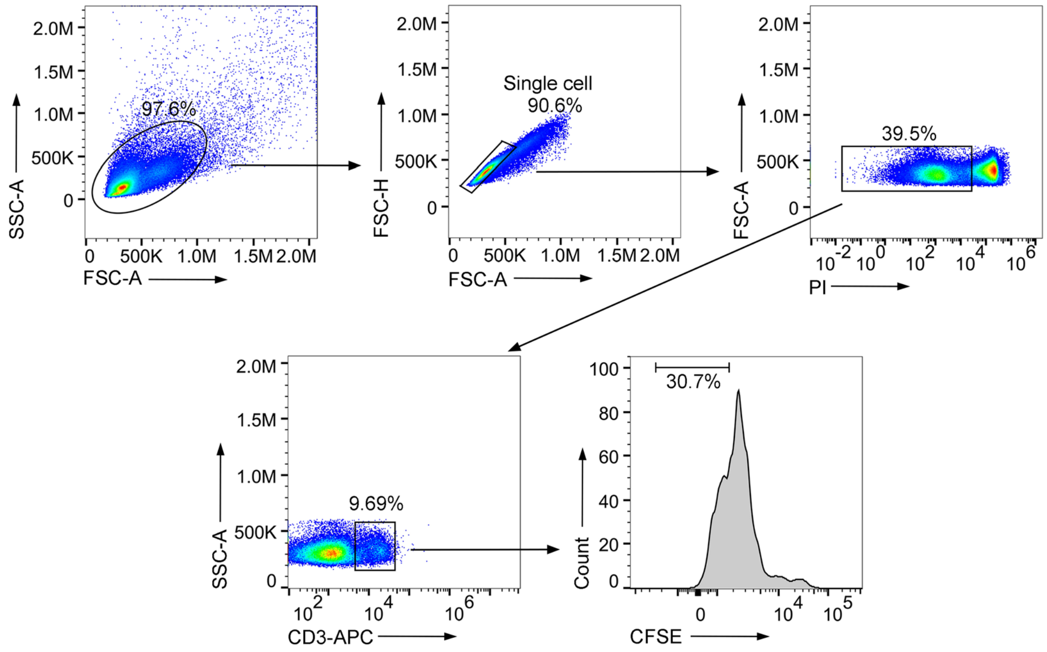

Supplementary Figure 42. Gating strategy for flow cytometric analysis on the proliferation of T cells within the activated splenocytes (Fig. 2f, Supplementary Fig. 9 and Supplementary Fig. 10).

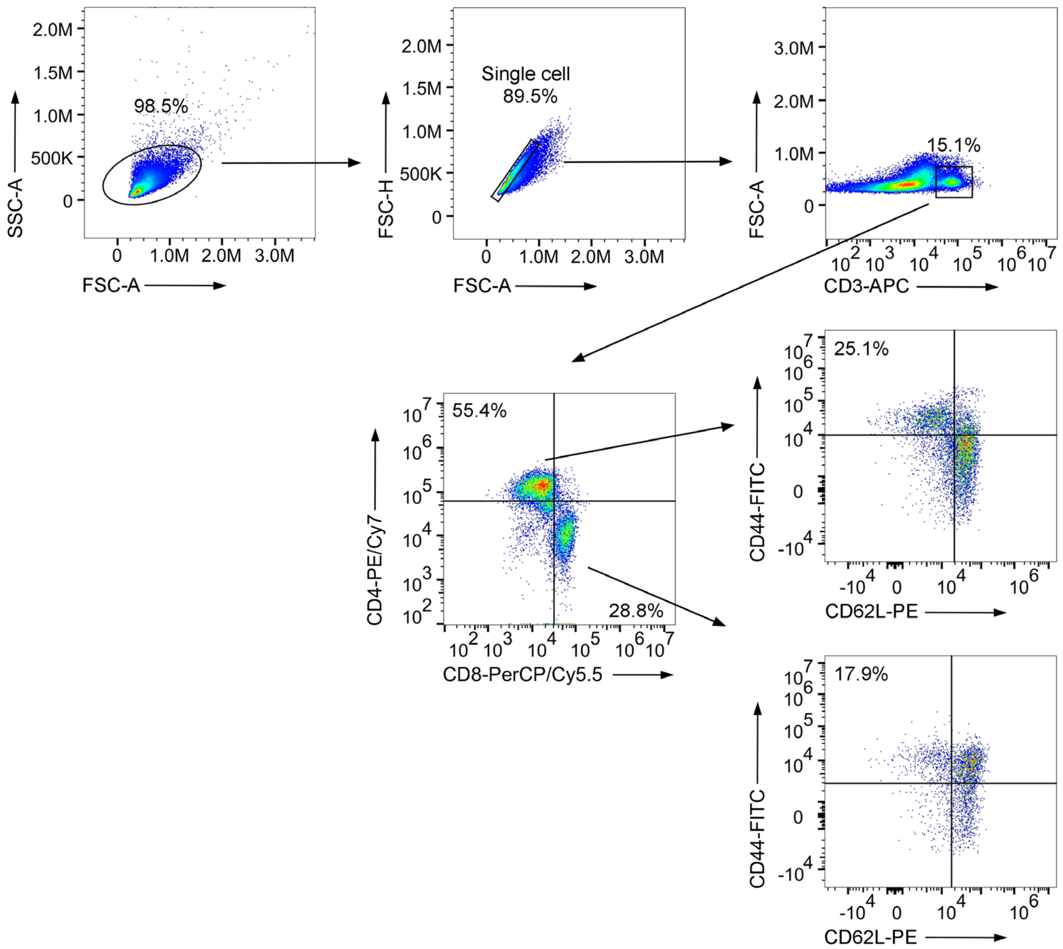

Supplementary Figure 43. Gating strategy for flow cytometric analysis on the percentage of cell population in the CD8$^+$ and CD4$^+$ T cells in the activated splenocytes (Supplementary Fig. 11a and Supplementary Fig. 11b).

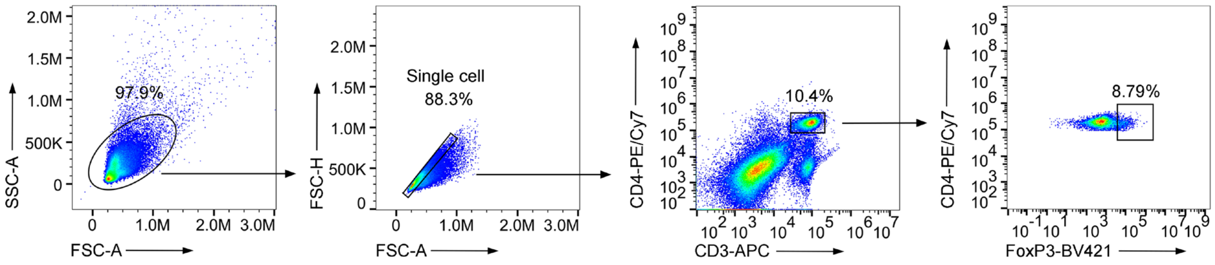

Supplementary Figure 44. Gating strategy for flow cytometric analysis on the percentage of Treg cells in $CD4^+$ T cells in the activated splenocytes (Supplementary Fig. 11c).

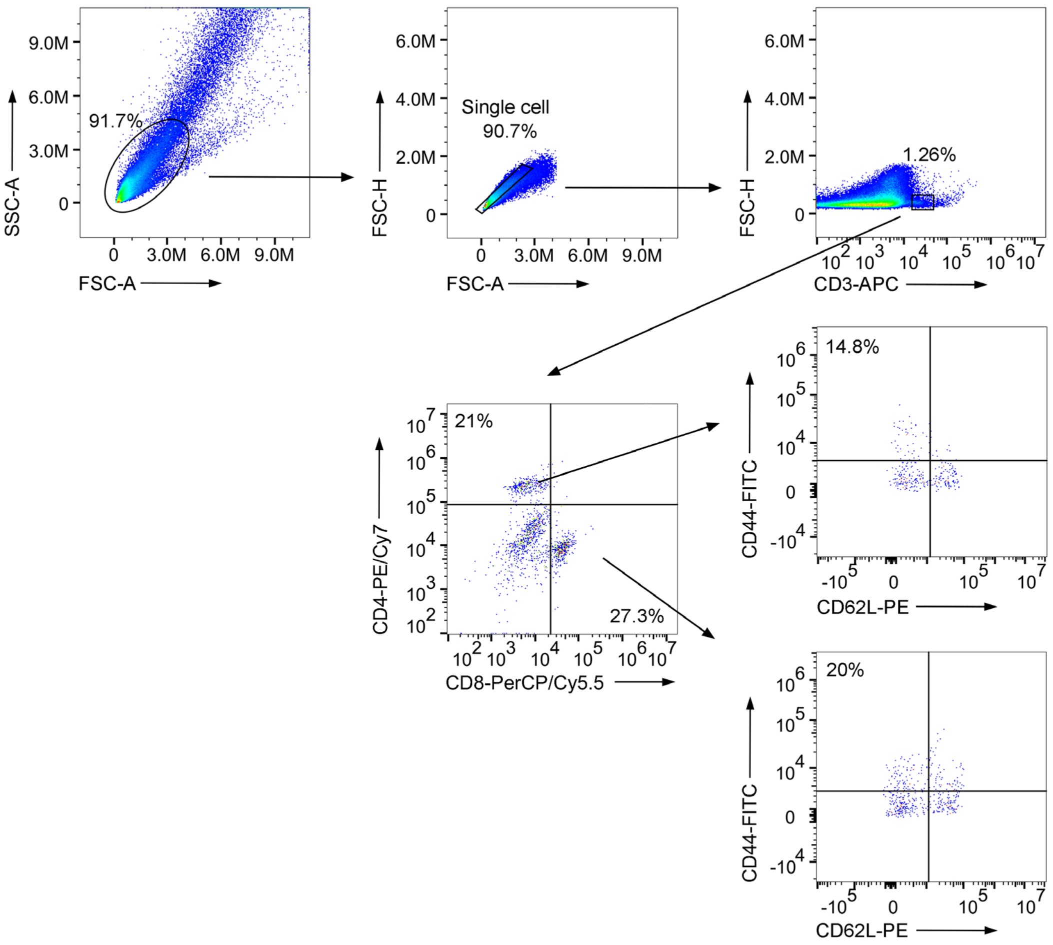

Supplementary Figure 45. Gating strategy for flow cytometric analysis on the percentage of cell population in the CD8^{+} and CD4^{+} T cells in the transplanted kidney (Fig. 3l, Fig. 3m, Supplementary Fig. 21a and Supplementary Fig. 21b).

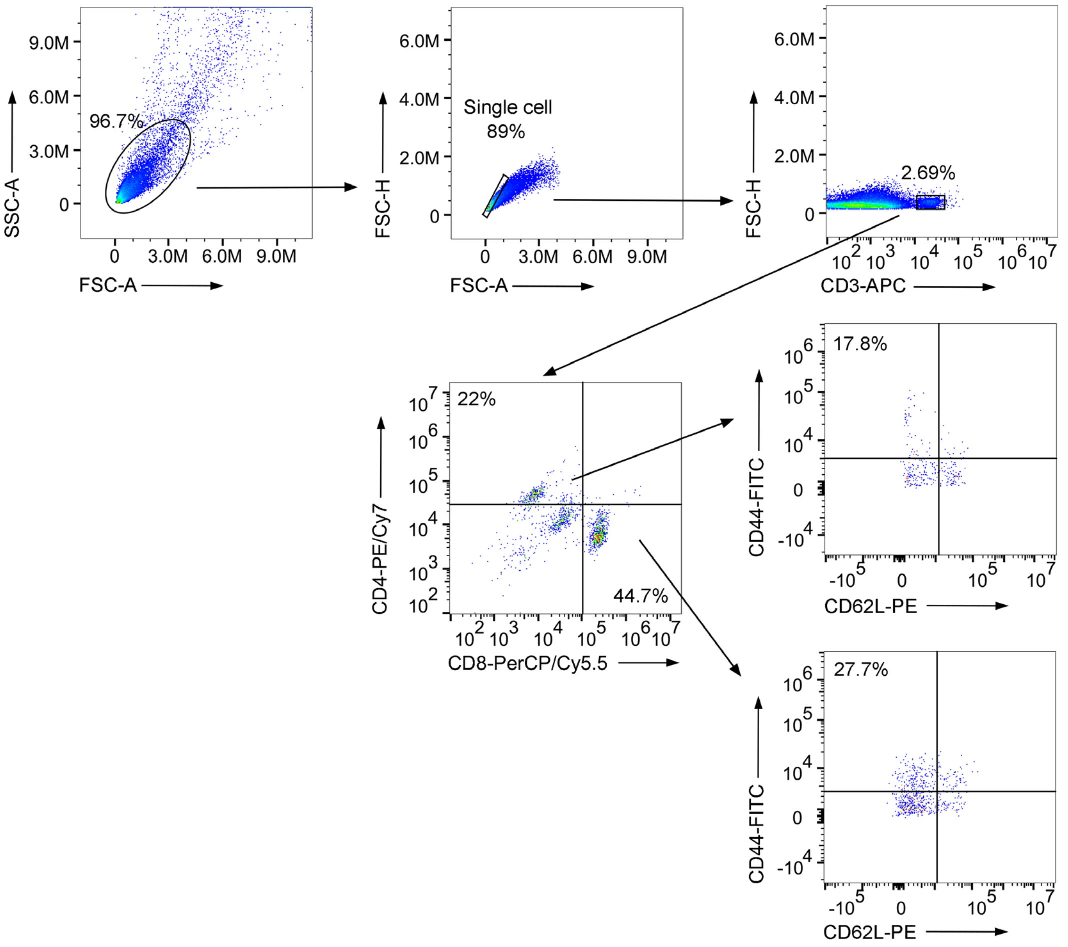

Supplementary Figure 46. Gating strategy for flow cytometric analysis on the percentage of cell population in the $CD8^+$ and $CD4^+$ T cells in skin (Fig. 5b, Fig. 5c, Supplementary Fig. 34a and Supplementary Fig. 34b).

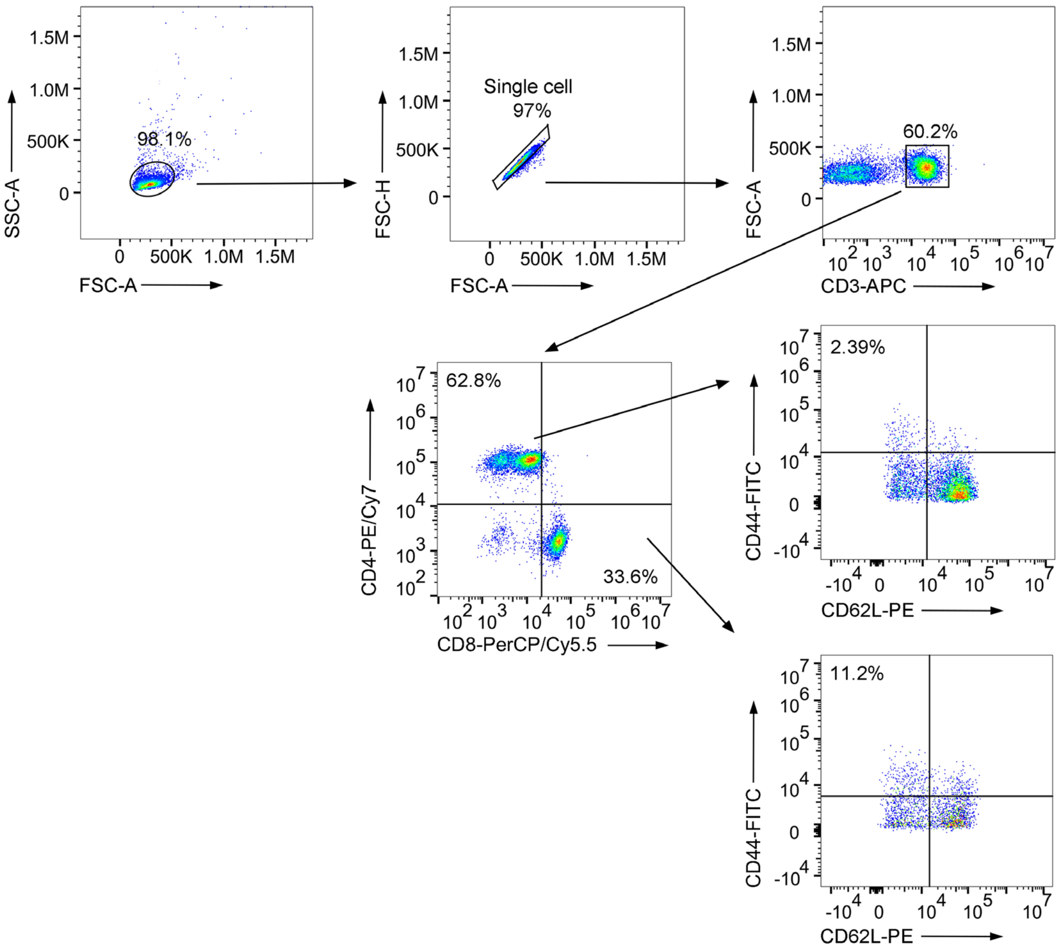

Supplementary Figure 47. Gating strategy for flow cytometric analysis on the percentage of cell population in the CD8^{+} and CD4^{+} T cells in KDLN/SDLN (Fig. 5e, Fig. 5f, Supplementary Fig. 24a, Supplementary Fig. 24b, Supplementary Fig. 35a and Supplementary Fig. 35b).

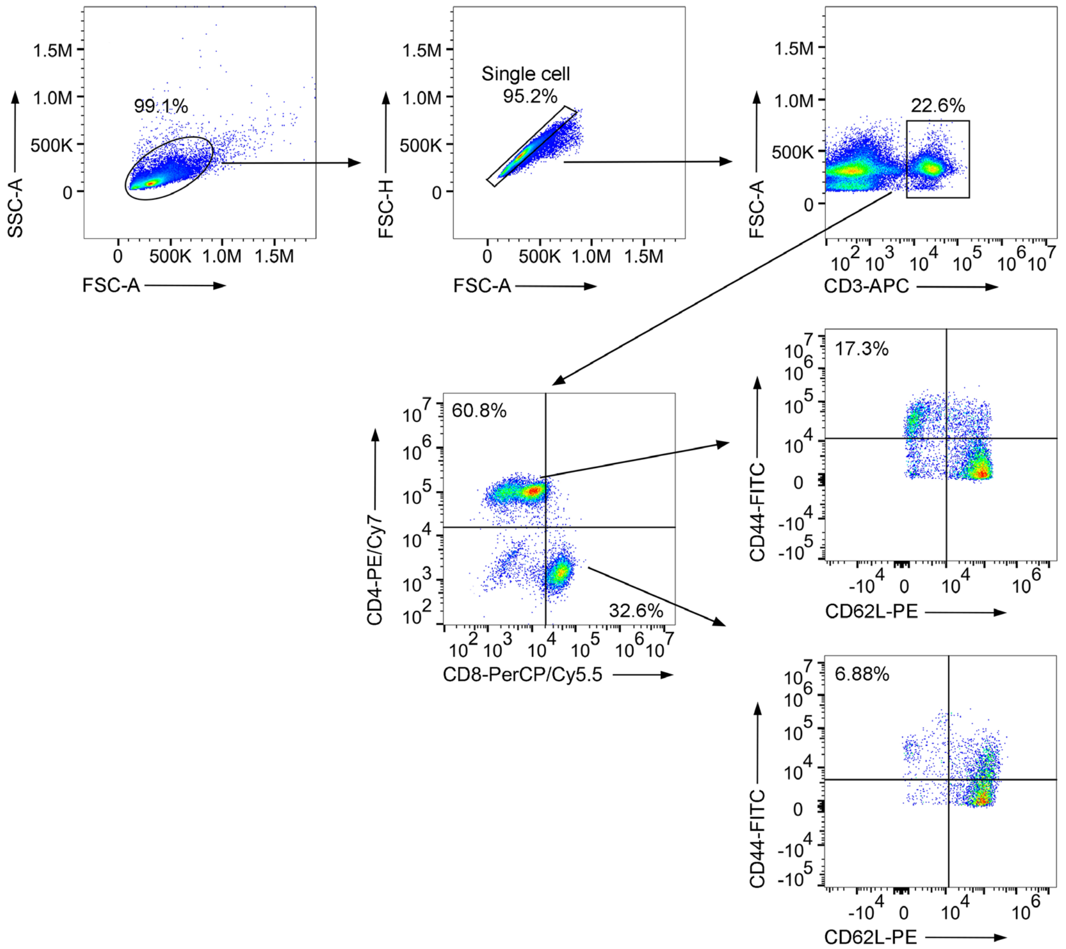

Supplementary Figure 48. Gating strategy for flow cytometric analysis on the percentage of cell population in the $CD8^+$ and $CD4^+$ T cells in spleen (Fig. 5h, Fig. 5i, Supplementary Fig. 25a, Supplementary Fig. 25b, Supplementary Fig. 36a and Supplementary Fig. 36b).

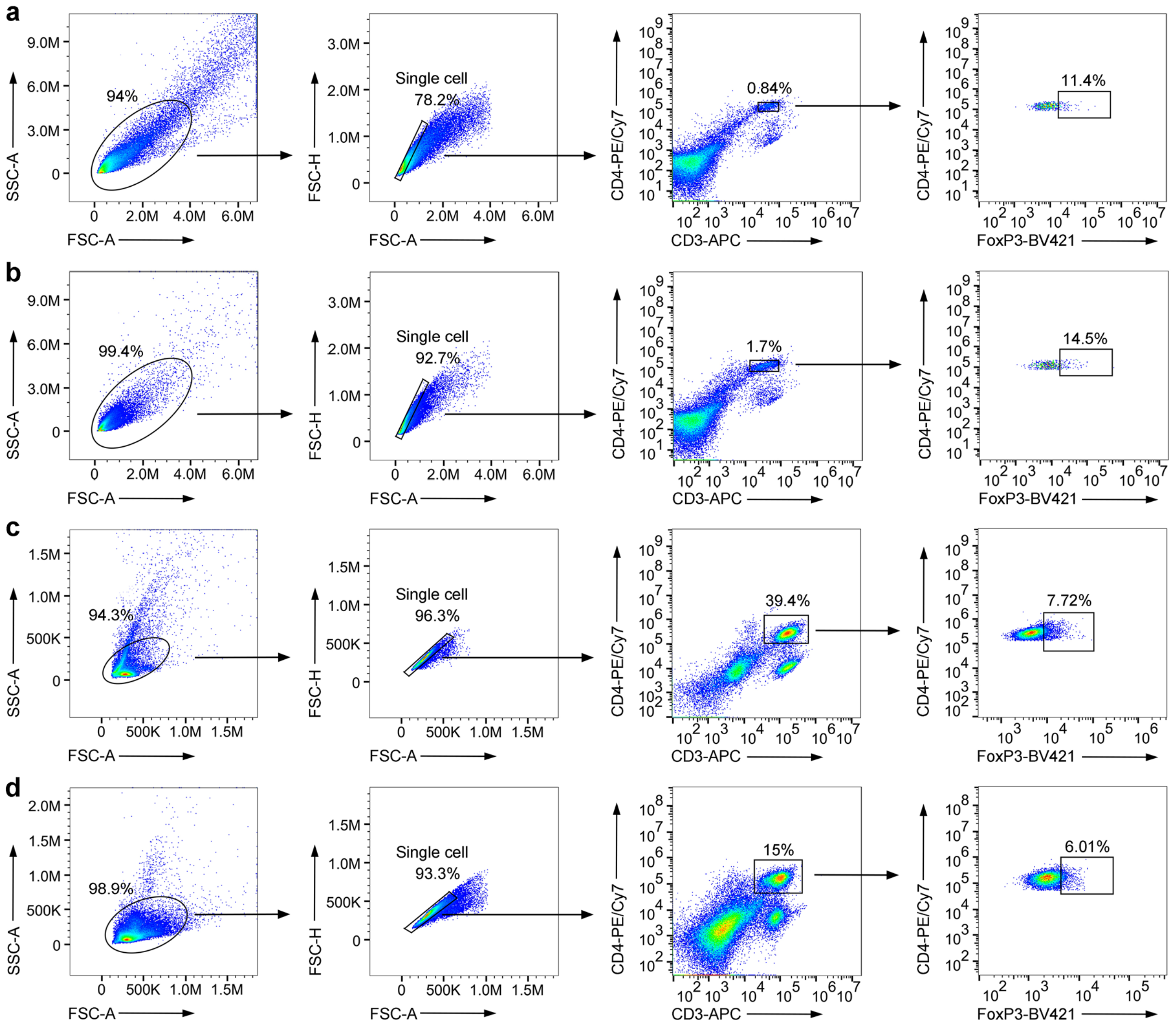

Supplementary Figure 49. Gating strategy for flow cytometric analysis on the percentage of Treg cells in CD4$^+$ T cells in the transplanted kidney (**a**), skin graft (**b**), KDLN/SDLN (**c**) and spleen (**d**) (Fig. 3n, Fig. 5d, Fig. 5g, Fig. 5j, Supplementary Fig. 21c, Supplementary Fig. 24c, Supplementary Fig. 25c, Supplementary Fig. 34c, Supplementary Fig. 35c and Supplementary Fig. 36c).

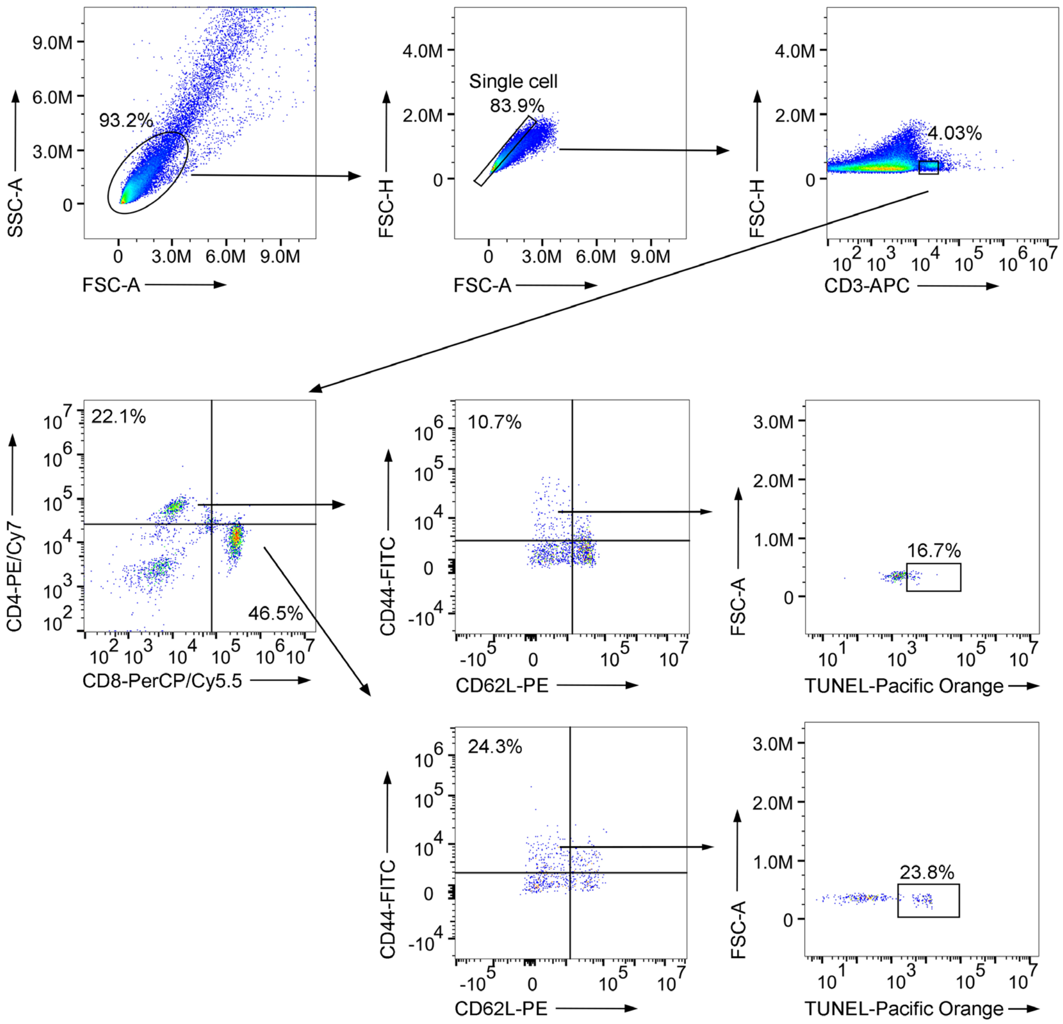

Supplementary Figure 50. Gating strategy for flow cytometric analysis on the percentage of TUNEL$^+$ cell population in the CD8$^+$ and CD4$^+$ Teff cells in the kidney (Supplementary Fig. 22).

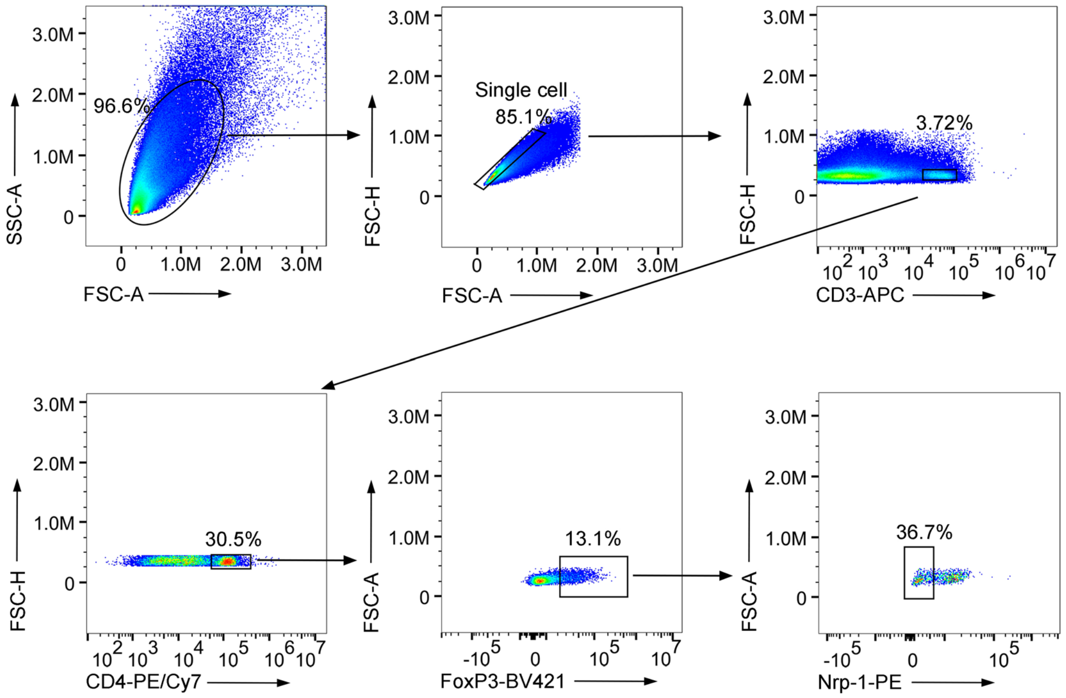

Supplementary Figure 51. Gating strategy for flow cytometric analysis on the percentage of iTreg ($CD4^+FoxP3^+Nrp\text{-}1^-$) cell population in the Treg cells in the transplanted kidney (Supplementary Fig. 23).

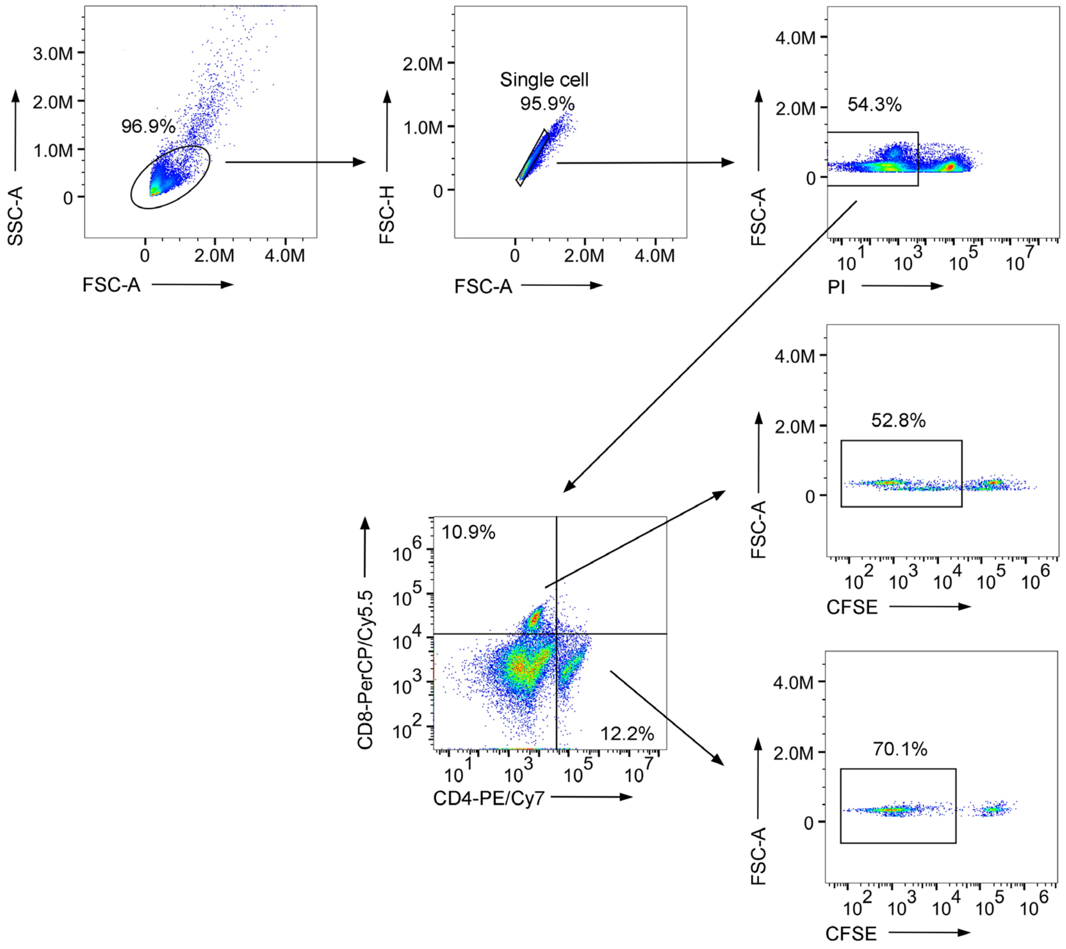

Supplementary Figure 52. Gating strategy for flow cytometric analysis on proliferative response of CD8$^+$ and CD4$^+$ T cells in spleen (Fig. 4g, Fig. 4h and Supplementary Fig. 29).
