# Supplementary material for: Immuno-protective vesicle-crosslinked hydrogel for allogenic transplantation
Source: Nat Commun. 2024 Jun 18;15:5176. doi: 10.1038/s41467-024-49135-x (PMC11189436; doi:10.1038/s41467-024-49135-x)
Supplement: Supplementary file 3 — Reporting Summary [file 41467_2024_49135_MOESM3_ESM.pdf]

nature portfolio

Corresponding author(s): Ran Mo

Last updated by author(s): Apr 23, 2024

Reporting Summary

Nature Portfolio wishes to improve the reproducibility of the work that we publish. This form provides structure for consistency and transparency in reporting. For further information on Nature Portfolio policies, see our Editorial Policies and the Editorial Policy Checklist.

Statistics

For all statistical analyses, confirm that the following items are present in the figure legend, table legend, main text, or Methods section.

n/a	Confirmed	
☐	☒	The exact sample size (*n*) for each experimental group/condition, given as a discrete number and unit of measurement
☐	☒	A statement on whether measurements were taken from distinct samples or whether the same sample was measured repeatedly
☐	☒	The statistical test(s) used AND whether they are one- or two-sided<br>*Only common tests should be described solely by name; describe more complex techniques in the Methods section.*
☒	☐	A description of all covariates tested
☐	☒	A description of any assumptions or corrections, such as tests of normality and adjustment for multiple comparisons
☐	☒	A full description of the statistical parameters including central tendency (e.g. means) or other basic estimates (e.g. regression coefficient) AND variation (e.g. standard deviation) or associated estimates of uncertainty (e.g. confidence intervals)
☐	☒	For null hypothesis testing, the test statistic (e.g. *F*, *t*, *r*) with confidence intervals, effect sizes, degrees of freedom and *P* value noted<br>*Give P values as exact values whenever suitable.*
☒	☐	For Bayesian analysis, information on the choice of priors and Markov chain Monte Carlo settings
☒	☐	For hierarchical and complex designs, identification of the appropriate level for tests and full reporting of outcomes
☒	☐	Estimates of effect sizes (e.g. Cohen's *d*, Pearson's *r*), indicating how they were calculated

Our web collection on statistics for biologists contains articles on many of the points above.

Software and code

Policy information about availability of computer code

Data collection	Olympus FV31S-SW software, Beckman CytExpert 2.4 software, BD FACSDiva software, Tecan i-control software, PerkinElmer Living Image software, Microsoft Excel.
Data analysis	GraphPad Prism 8.0, FlowJo 10, Living Image, Microsoft Excel.

For manuscripts utilizing custom algorithms or software that are central to the research but not yet described in published literature, software must be made available to editors and reviewers. We strongly encourage code deposition in a community repository (e.g. GitHub). See the Nature Portfolio guidelines for submitting code & software for further information.

Data

Policy information about availability of data

All manuscripts must include a data availability statement. This statement should provide the following information, where applicable:

- Accession codes, unique identifiers, or web links for publicly available datasets
- A description of any restrictions on data availability
- For clinical datasets or third party data, please ensure that the statement adheres to our policy

All the data supporting the findings of this study are available within the article and supplementary information. Source data are provided with this paper.

Research involving human participants, their data, or biological material

Policy information about studies with human participants or human data. See also policy information about sex, gender (identity/presentation), and sexual orientation and race, ethnicity and racism.

Reporting on sex and gender	Our studies do not involve human research participants.
Reporting on race, ethnicity, or other socially relevant groupings	n/a
Population characteristics	n/a
Recruitment	n/a
Ethics oversight	n/a

Note that full information on the approval of the study protocol must also be provided in the manuscript.

Field-specific reporting

Please select the one below that is the best fit for your research. If you are not sure, read the appropriate sections before making your selection.

☒ Life sciences ☐ Behavioural & social sciences ☐ Ecological, evolutionary & environmental sciences

For a reference copy of the document with all sections, see nature.com/documents/nr-reporting-summary-flat.pdf

Life sciences study design

All studies must disclose on these points even when the disclosure is negative.

Sample size	Sample size for each experiment was specified in the figure captions.
Data exclusions	No data were excluded.
Replication	Experiments were repeated and experimental findings were reproducible.
Randomization	Randomization was applied to each experimental group.
Blinding	No formal blinding was used.

Reporting for specific materials, systems and methods

We require information from authors about some types of materials, experimental systems and methods used in many studies. Here, indicate whether each material, system or method listed is relevant to your study. If you are not sure if a list item applies to your research, read the appropriate section before selecting a response.

Materials & experimental systems

n/a	Involved in the study	
☐	☒	Antibodies
☐	☒	Eukaryotic cell lines
☒	☐	Palaeontology and archaeology
☐	☒	Animals and other organisms
☒	☐	Clinical data
☒	☐	Dual use research of concern
☒	☐	Plants

Methods

n/a	Involved in the study	
☒	☐	ChIP-seq
☐	☒	Flow cytometry
☒	☐	MRI-based neuroimaging

Antibodies

Antibodies used

APC-labelled anti-mouse CD34 antibody (BioLegend, HM34, 128611)
PE-labelled anti-mouse CD45 antibody (BioLegend, I3/2.3, 147711)
APC-labelled anti-mouse CD90.2 antibody (BioLegend, 30-H12, 105311)
PerCP/Cy5.5-labelled anti-mouse/rat CD29 antibody (BioLegend, HMβ1-1, 102227)
APC-labelled anti-mouse CD3 antibody (BioLegend, 17A2, 100236)
PE/Cy7-labelled anti-mouse CD4 antibody (BioLegend, GK1.5, 100422)

PerCP/Cy5.5-labelled anti-mouse CD8a antibody (BioLegend, 53-6.7, 100734)
FITC-labelled anti-mouse/human CD44 antibody (BioLegend, IM7, 103006)
PE-labelled anti-mouse CD62L antibody (BioLegend, MEL-14, 104407)
Brilliant Violet 421 (BV421)-labelled anti-mouse FoxP3 antibody (BioLegend, MF-14, 126419)
FITC-labelled anti-mouse PD1 antibody (BioLegend, 29F.1A12, 135213)
APC-labelled anti-mouse PD-L1 antibody (BioLegend, 10F.9G2, 124311)
FITC-labelled anti-mouse Fas antibody (BioLegend, SA367H8, 152605)
PE-labelled anti-mouse Neuropilin-1 (Nrp-1) antibody (BioLegend, 3E12, 145203)
PE-labelled anti-mouse FasL antibody (Invitrogen, MFL3, 12591181)
rabbit anti-mouse FasL antibody (Affinity, Polyclonal, AF0157)
rabbit anti-mouse PD-L1 antibody (Affinity, Polyclonal, DF6526)
HRP-labelled goat anti-rabbit IgG antibody (Proteintech, Polyclonal, SA00001-2)

Validation

All antibodies were verified by the supplier and each lot has been quality tested.

Eukaryotic cell lines

Policy information about cell lines and Sex and Gender in Research

Cell line source(s)

MSCs were isolated from the bone marrow of the BALB/c mice.
Murine pancreatic islets were isolated from the C57BL/6 mice.
T cells were sorted from the splenocytes of the BALB/c mice.
RBCs and PLTs were collected from the blood of the BALB/c mice.

Authentication

All of the cells are primary cells harvested from mice and were not further authenticated.

Mycoplasma contamination

All the cells were routinely examined for mycoplasma contamination. No mycoplasma contamination was found.

Commonly misidentified lines
(See ICLAC register)

No commonly misidentified cell lines were used.

Animals and other research organisms

Policy information about studies involving animals; ARRIVE guidelines recommended for reporting animal research, and Sex and Gender in Research

Laboratory animals

BALB/c and C57BL/6 mice (female, ~20g) were provided by the Comparative Medicine Centre of Yangzhou University. C3H mice (female, ~20g) were purchased from the Beijing Vital River Laboratory Animal Technology Co., Ltd. Foxp3/DTR mice (female, ~20g) were obtained from the Shanghai Model Organisms Center, Inc.

Wild animals

The study did not involve wild animals.

Reporting on sex

This information has not been collected.

Field-collected samples

The study did not involve samples collected from field.

Ethics oversight

All the animals were treated according to the Guide for Care and Use of Laboratory Animals, approved by the Animal Experimentation Ethics Committee of China Pharmaceutical University.

Note that full information on the approval of the study protocol must also be provided in the manuscript.

Plants

Seed stocks

Our studies do not involve Plants.

Novel plant genotypes

n/a

Authentication

n/a

Flow Cytometry

Plots

Confirm that:

- [x] The axis labels state the marker and fluorochrome used (e.g. CD4-FITC).
- [x] The axis scales are clearly visible. Include numbers along axes only for bottom left plot of group (a 'group' is an analysis of identical markers).
- [x] All plots are contour plots with outliers or pseudocolor plots.
- [x] A numerical value for number of cells or percentage (with statistics) is provided.

Methodology

Sample preparation	All the samples were prepared as described in the Methods.
Instrument	Beckman CytoFlex S flow cytometer for analysis; BD FACSAria II flow cytometer for cell sorting.
Software	Beckman CytExpert 2.4 software and BD FACSDiva software were used to collect the data. FlowJo 10 software was used to analyse the data.
Cell population abundance	At least 10,000 cells were used for flow cytometric analysis, while at least 1,000,000 cells were used for flow cytometric sorting.
Gating strategy	Gating strategy was provided in the Supplementary Information.

- [x] Tick this box to confirm that a figure exemplifying the gating strategy is provided in the Supplementary Information.
